# In vitro selection of ribozyme ligases that use prebiotically plausible 2-aminoimidazole–activated substrates

**DOI:** 10.1073/pnas.1914367117

**Published:** 2020-03-02

**Authors:** Travis Walton, Saurja DasGupta, Daniel Duzdevich, Seung Soo Oh, Jack W. Szostak

**Affiliations:** ^a^Howard Hughes Medical Institute, Massachusetts General Hospital, Boston, MA 02114;; ^b^Department of Molecular Biology, Center for Computational and Integrative Biology, Massachusetts General Hospital, Boston, MA 02114;; ^c^Department of Materials Science and Engineering, Pohang University of Science and Technology, 37673 Pohang, Gyeongbuk, South Korea

**Keywords:** ribozymes, ligation, prebiotic

## Abstract

The hypothesized central role of RNA in the origin of life suggests that RNA propagation predated the advent of complex protein enzymes. A critical step of RNA replication is the template-directed synthesis of a complementary strand. Two experimental approaches have been extensively explored in the pursuit of demonstrating protein-free RNA synthesis: template-directed nonenzymatic RNA polymerization using intrinsically reactive monomers and ribozyme-catalyzed polymerization using more stable substrates such as biological 5′-triphosphates. Despite significant progress in both approaches in recent years, the assembly and copying of functional RNA sequences under prebiotic conditions remains a challenge. Here, we explore an alternative approach to RNA-templated RNA copying that combines ribozyme catalysis with RNA substrates activated with a prebiotically plausible leaving group, 2-aminoimidazole (2AI). We applied in vitro selection to identify ligase ribozymes that catalyze phosphodiester bond formation between a template-bound primer and a phosphor-imidazolide–activated oligomer. Sequencing revealed the progressive enrichment of 10 abundant sequences from a random sequence pool. Ligase activity was detected in all 10 RNA sequences; all required activation of the ligator with 2AI and generated a 3′-5′ phosphodiester bond. We propose that ribozyme catalysis of phosphodiester bond formation using intrinsically reactive RNA substrates, such as imidazolides, could have been an evolutionary step connecting purely nonenzymatic to ribozyme-catalyzed RNA template copying during the origin of life.

## Significance

Current models for the origin of life include an earlier period when prebiotic chemistry dictated the nonenzymatic copying of RNA polymers, followed by a period of ribozyme-catalyzed reactions using nucleoside-5′-triphosphate (NTP) substrates. Our study addresses the transition from nonenzymatic to ribozyme-catalyzed RNA template copying by determining whether ribozymes could have used substrates activated with 2-aminoimidazole, a prebiotically plausible leaving group, instead of NTPs. We identify ligase ribozymes that use RNA substrates activated with 2-aminoimidazole, thereby proving that ribozyme catalysis is compatible with the reactants of nonenzymatic template-directed ligation. This work suggests that the transition from nonenzymatic to ribozyme-catalyzed RNA replication could have involved ribozymes that utilize inherently reactive RNA substrates.

The RNA world hypothesis proposes a central role for RNA as both a catalytic and an informational polymer during the emergence of life. Evidence for the RNA world includes a variety of functional RNAs, such as ribozymes and riboswitches, as well as the prebiotically plausible syntheses of nucleotides (1, 2). An important tenet of the RNA world hypothesis is that informational and functional RNA polymers must have preceded complex protein enzymes, as exemplified by the RNA-catalyzed synthesis of proteins inside the catalytic core of the ribosome (3). However, the antecedence of functional RNA raises fundamental questions regarding the formation and replication of RNA polymers during the origin of life, when protein enzymes were absent. Under nonenzymatic reaction conditions, the formation of phosphodiester bonds using biological nucleoside-5′-triphosphates (NTPs) is slower than the hydrolysis of phosphodiester linkages under identical conditions, which would severely limit the accumulation of RNA polymers (4, 5).

Experimental studies have instead utilized a variety of intrinsically reactive monomers and oligomers to form both random single-stranded polymers and templated double-stranded RNA products (6–8). In particular, imidazoles have been extensively studied as the leaving group of RNA monomers called nucleoside-5′-phosphor-imidazolides (9, 10). Recent work has demonstrated that 2-aminoimidazole (2AI) shares a common synthetic pathway with prebiotic nucleotide synthesis, and 2AI-activated monomers enhance the rate and extent of nonenzymatic template-directed polymerization (11, 12). 2AI-activated RNA monomers can be generated by a prebiotically relevant chemical pathway involving nucleotides, isocyanides, and aldehydes, in the presence of free 2AI, and the repeated addition of isocyanide results in multiple cycles of monomer reactivation and spontaneous hydrolysis (13–15). These recent findings are particularly relevant because isocyanide is produced by a variation of the ferrocyanide chemistry that creates cyanamide, a precursor of RNA nucleotides and 2AI (1, 15). Thus, 2AI-activated RNA nucleotides, and the ensuing oligomeric products, provide a reasonable set of reactants to experimentally model nonenzymatic RNA copying reactions during the origin of life.

Due to seemingly inherent limitations associated with nonenzymatic sequence copying, the emergence of macromolecular catalysts that improve the yield and fidelity of RNA polymerization would have likely provided a selective advantage for early cellular life (16). RNA catalysts are thought to have played an important role during the origin of life, and ribozyme-catalyzed RNA polymerization provides an attractive solution to the problem of protein-free RNA copying. In vitro selection has successfully identified and evolved ribozymes that catalyze template-directed ligation and polymerization reactions using RNA monomers and oligomers activated with the biologically ubiquitous 5′-triphosphate group (17–19). The potential of polymerase and ligase ribozymes for self-replication and self-assembly strongly suggests that such ribozymes could have played an important role in RNA replication during the origin of life (20–23). However, the sequence length and complexity of in vitro-selected polymerase ribozymes vastly exceed the currently understood limits of nonenzymatic RNA synthesis, a process that must have preceded enzymatic assembly. One possible explanation is that shorter functional RNAs with limited catalytic abilities could have played a role in the transition from chemical polymerization reactions to ribozyme-catalyzed copying. For example, nonenzymatic template-directed RNA polymerization is catalyzed by trinucleotide “helper” RNAs that help preorganize the site of primer extension (24–26). In addition, remarkably short (5–30 nt) ribozymes and aptamers have been discovered, although outside the context of RNA copying (27, 28).

Since functional RNAs must have emerged from nonenzymatic processes, we reasoned that during the origin of life, polymerase and ligase ribozymes might have used intrinsically reactive substrates such as imidazolides instead of NTP monomers. Imidazolide RNA monomers and oligomers react in the absence of enzymes, so even relatively short functional sequences might be expected to significantly enhance the rate, extent, or fidelity of template-directed RNA copying. In this hypothetical scenario, both nonenzymatic and ribozyme-catalyzed phosphodiester bond formation would operate simultaneously, with rate-limiting steps of nonenzymatic replication being the subjects of ribozyme evolution. However, a previous selection to isolate ligase ribozymes that utilize imidazolide substrates yielded an unanticipated catalytic activity, namely formation of a 5′-5′ tetraphosphate linkage instead of the desired 3′-5′ phosphodiester linkage (29). This 5′-5′ linkage is not typically desirable for copying sequence information and casts doubt over the possible connection between nonenzymatic and ribozyme-catalyzed copying reactions.

Here, we present the identification of ligase ribozymes that catalyze template-directed formation of a phosphodiester linkage using a 5′-phosphoro-2-aminoimidazolide oligomer substrate. We isolated ribozyme ligases through in vitro selection using a biotinylated 2AI-activated ligator as bait. We characterized 10 highly enriched sequences, all of which displayed ligase activity. Our results highlight the potential of combining prebiotic activation chemistry with in vitro selection to further advance RNA-catalyzed RNA polymerization. In addition, the identification of imidazolide ligase ribozymes supports a hypothesized transitional stage whereby ribozyme-catalyzed reactions coexisted with nonenzymatic reactions.

## Results

### Library Design for In Vitro Selection of Imidazolide Ligase Ribozymes.

To investigate the compatibility of nonenzymatic and ribozyme-catalyzed RNA synthesis, we sought to identify ribozymes that catalyze the template-directed ligation of a 2AI-activated substrate. Due to fundamental differences in the underlying chemical mechanism, the rate of nonenzymatic ligation is slower than polymerization for imidazolide RNA reactants under typical reaction conditions with millimolar concentrations of activated monomers (25, 30). This makes ligation of 2AI-activated substrates a conspicuous target for ribozyme catalysis, as they are potential rate-limiting steps for the assembly or copying of longer functional RNA sequences. In a ligation reaction, a hydroxyl group from the 3′ terminus of an RNA primer attacks the 2AI-activated phosphate group of an RNA ligator, displacing the 2AI leaving group and forming a single phosphodiester linkage. Ligation reactions using 5′-triphosphate–activated substrates have been extensively used to identify ribozymes through in vitro selection (17, 31, 32), and we reasoned that a similar strategy could be used to select ribozymes that use imidazolide substrates. Ligation offers several advantages that promote control over the selection procedure. For example, ligation can be used to physically link an affinity tag to a functional sequence in the RNA library, aiding in the enrichment of active sequences.

**Fig. 1.**
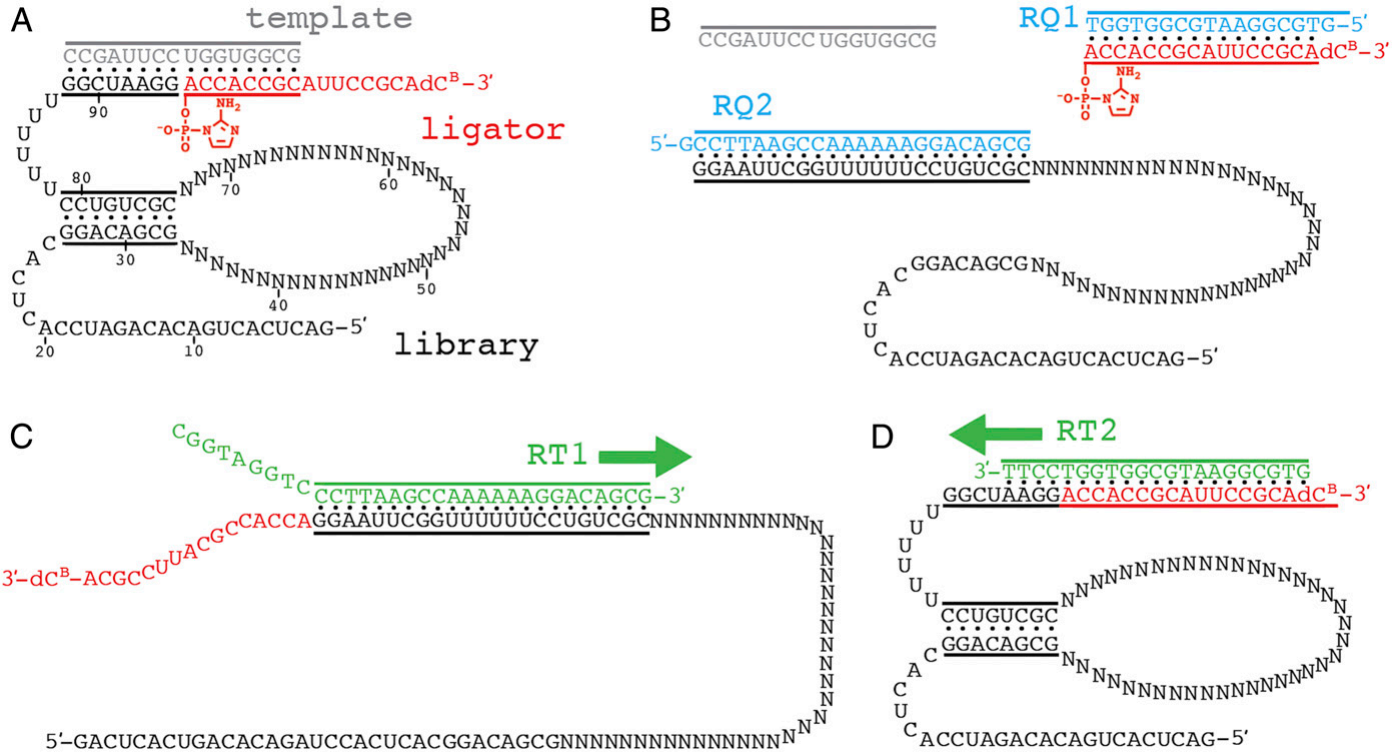
Experimental strategy to select for ribozymes that catalyze ligation of 2-aminoimidazolide–activated oligonucleotides. (*A*) Design of the selection library. The 95-nt RNA library (black) consists of a 25-nt constant region at the 5′ end, an 8-bp stem enclosing a 40-nt loop of random sequences, a six-uridine linker, and an 8-nt primer region at the 3′ end. The primer binds a 16-nt template (gray) and reacts with a 17-nt ligator (red) that features 5′-phosphor-2-aminoimidazolide activation and a 3′ terminal biotinylated deoxycytidine nucleotide (dC^B^). (*B*) The ligation reaction is quenched by annealing with a large excess of cDNA strands RQ1 and RQ2 (blue) and chelators. (*C*) After affinity purification of ligated RNA molecules, the selected sequences are reverse transcribed with the DNA primer RT1, which is complementary to the 3′ end of the RNA library. (*D*) In rounds 4–8, a more selective DNA primer was used for reverse transcription, which is complementary to the last 4 nt of the RNA library and all 17 nt of the ligator sequence.

We designed a 95-nt RNA library that is characterized by a partially random stem loop structure physically attached to a template-bound primer (Fig. 1*A*, black). If the particular stem loop catalyzes ligation of a separate 2AI-activated ligator strand (Fig. 1*A*, red), it will be selected by affinity purification, reverse transcribed, and amplified by PCR. In order to promote secondary structure formation and encourage RNA folding, the RNA library incorporates a predefined 8-bp stem that closes a 40-nt random loop. Attached to the 3′ end of the stem-loop structure is a six-uridine (U6) RNA linker and an 8-nt primer region. This primer region is bound through eight Watson–Crick base pairs to a separate template strand (Fig. 1*A*, gray). The template strand also binds a separate ligator strand that is activated as a 5′-phosphor-2-aminoimidazolide (Fig. 1*A*, red). The ligator strand is 17 nt long, with the 3′ terminus being a biotinylated deoxycytidine (dC^B^) nucleotide, and the rest being standard RNA nucleotides. Only 8 of 17 nt of the ligator are bound by the template, creating a 9-bp overhang at the 3′ end of the ligator. This overhang was incorporated into the ligator sequence to promote strand displacement of the ligator from unreacted complexes during reaction quenching (Fig. 1*B*). Lastly, the selection library also incorporates a 25-nt constant region at the 5′ end that is used for PCR amplification of selected sequences. This region is therefore not subject to heritable mutation, but as part of the RNA library, it may be required for proper folding of some functional sequences.

### Selection of Ligase Ribozymes That Use 2AI-Activated Substrates.

The initial RNA pool of 1.1 nmol (∼6.6 × 10^14^ sequences) was prepared by transcription from 2.4 nmol of solid-phase synthesized DNA; the RNA was then digested with Ava-II to generate homogenous 3′ ends with free hydroxyl groups (33). For the first round of selection, the RNA library was annealed with a slight excess of template and activated ligator strands, and then the ligation reaction was initiated by the addition of 20 mM MgCl_2_. After 2 h of incubation, the reaction was quenched with EDTA and an excess of two cDNA strands targeting the ligator and the 3′ end of the library (Fig. 1*B*). Ligated RNA strands were purified with magnetic streptavidin-coated beads, with nonligated RNA complexes removed by washing the beads with 8 M urea and 25 mM NaOH. Ligated strands were eluted by heating the beads at 65 °C in 95% formamide. Recovered material was then reverse transcribed and amplified by PCR using primers that reintroduce the T7 promoter.

In subsequent rounds, the stringency of the selection protocol was increased by shortening the incubation time of the ligation reaction (*SI Appendix*, Table S1). For rounds 2 and 3, the reaction time was halved to 60 min and 30 min, respectively, and further reduced to 10 min for rounds 6–8. Beginning in the fourth round, the selection protocol incorporated a reverse transcription primer that was complementary to only the ligator sequence, whereas previously, this primer was complementary to the 3′ end of the library itself (Fig. 1 *C* and *D*). We reasoned that this primer would bias the reverse transcription step against any sequences that were nonspecifically retained during affinity purification. Since reverse transcription begins with a DNA primer bound to the 3′ end of an RNA template, we also expected that ribozymes using alternative ligation sites (e.g., a 5′-5′ ligase) would be disfavored by the new reverse transcription primer.

**Fig. 2.**
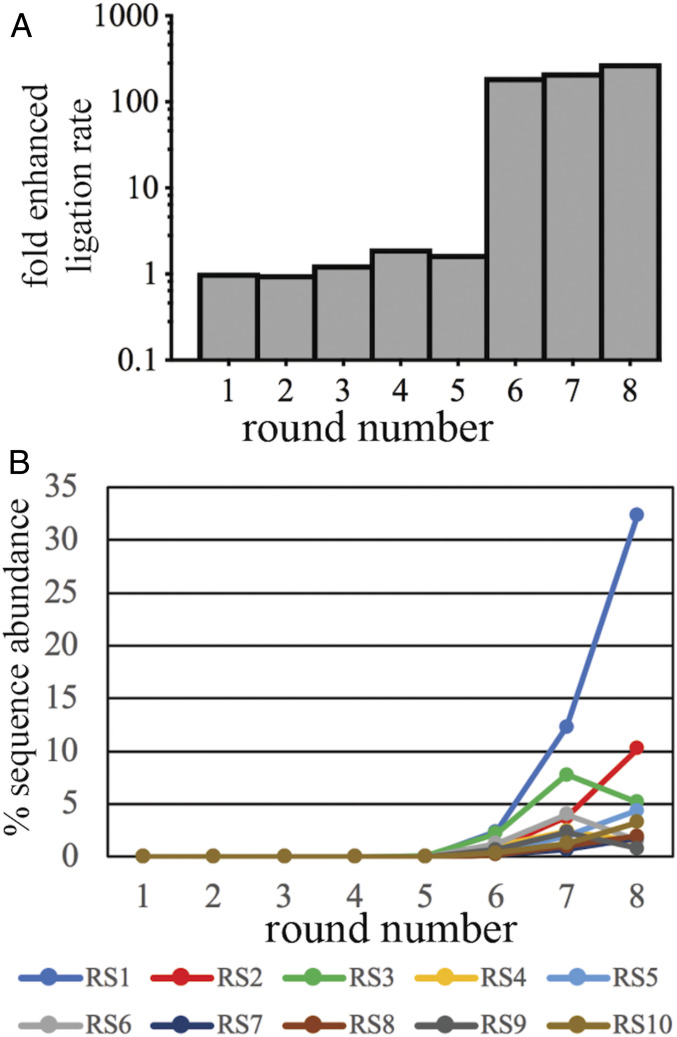
Enrichment of ligase activity through eight rounds of selection. (*A*) The RNA pools after each round of selection were assayed in a ligation reaction to determine the fold enhancement of the ligation rate relative to the naive pool. (*B*) Percentage sequence abundances of RS1–RS10 for all eight rounds as determined by deep sequencing.

After each round of selection, the RNA pool was analyzed for ligase activity in a reaction with the 2AI-activated ligator RNA strand. Relative to the baseline nonenzymatic rate of imidazolide ligation, significant ligase activity was observed after round 6 (Fig. 2*A*). Encouraged by this result, we further increased the stringency of the selection protocol by decreasing the reaction time and the MgCl_2_ concentration for the final rounds (*SI Appendix*, Table S1). After the eighth round of selection, the enhancement of the ligation rate had increased by a factor of ∼200. We also tested whether the putative ligase activity of the RNA pool depended upon the presence of the template strand. While ligation product was observed by 3 min when the template was included, omission of the template resulted in no detectable ligation product by 30 min (*SI Appendix*, Fig. S1). This result suggests that the most abundant functional sequences were catalyzing template-directed phosphodiester bond formation as intended.

### Identification of Functional Sequences.

Pleased with the catalytic activity observed in the RNA pool, we next characterized the abundance of distinct sequences in each of the RNA pools in order to identify potential ligase ribozymes. All eight rounds were sequenced in a multiplexed Illumina MiSeq run, and sequences that passed quality control were counted. In general, rounds 1–5 were more diverse and did not contain any individual sequence with greater than a 0.03% abundance. In rounds 6–8, the number of unique sequences per 1,000 reads decreased from 502 to 28, and a group of 10 sequences achieved greater than 1% abundance by the eighth round. Notably, a single sequence referred to as RS1 was the most abundant sequence from rounds 6 through 8, comprising 32% (202,551 counts) of the total reads of round 8 (Fig. 2*B* and Table 1). A point mutation of RS1 was observed at 1.8% abundance, and thus, we grouped related sequences to quantify abundances of clusters as well as individual sequences (*SI Appendix*, Table S2). This analysis revealed 64 clusters with >0.1% abundance in the round 8 pool, suggesting that our selection may have identified a variety of functional sequences that catalyze the ligation of a 5′-phosphoro-2-aminoimidazolide substrate. Multiple sequence alignment (34, 35) of the peak sequences in each cluster only revealed short stretches (6–10 nt) of perfect alignment between these sequences, further suggesting that these clusters were independently derived (*SI Appendix*, Fig. S2). We chose 10 independently derived sequences, each the most abundant of their respective cluster, for further characterization (Table 1).

**Table 1.** Sequences corresponding to the N40 loop region (positions 34–73 of the library) of enriched sequences RS1–RS10 identified by deep sequencing

	Loop sequence (5′ to 3′)	Round 8 abundance, %
RS1	GAAUGCUGCCAACCGUGCGGGCUAAUUGGCAGACUGAGCU	32
RS2	CCUAGCUAGCGCUGACUAGGACAGAUGAGCGGCGGAACCA	10
RS3	UUAGUGAAAUUGGUGCCCAAGCAGAGAAUUGGGAUAAAUC	5.2
RS4	UCAGUCGGAGUACCAGAGCGAUAGACGUCCCCGGAAGCCG	1.5
RS5	GAACCCUUAUCACAGUCGUGCGGAUUUGUAAGCCUAAGCG	4.4
RS6	CUGGCAAACACAGCGCGCUGUGUGUUAUGUGGGGCGGUCU	1.6
RS7	AAAAGUUUCGCUGAAUUGGACAGACCACCGCGUGAAGUGG	1.8
RS8	AGCCACUGCGGAAGACCUUAAGAGGUGUAAUUGCUCACCC	2.0
RS9	AAGCUCUCGCCAGCAAAAGAACAGACCGUCGAGGAAACGG	0.82
RS10	AGAGACCGUGAGCUUGCGGAAUGUUAGCAGAACAGAACUG	3.3

**Fig. 3.**
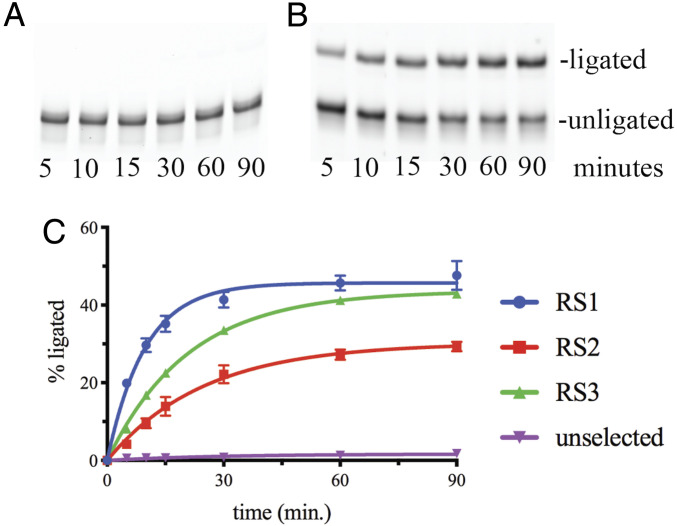
Validation of ligase activity for sequences RS1–RS3. Analysis of ligation reactions by 10% PAGE over the course of 90 min using unselected, naive RNA pool (*A*), and candidate ribozyme RS1 (*B*). (*C*) Quantification of ligation reactions in triplicate for the unselected RNA pool and for enriched RNA sequences RS1–RS3. Line indicates the nonlinear fit of the data to an exponential function.

To determine whether the enriched sequences were functional, we began individually testing these putative ligases in kinetic assays with the 2AI-activated ligator. Ligation was observed over a 90-min period and compared to a sample of the original unselected RNA pool (Fig. 3). For the unselected RNA pool, the ligation reaction is very slow, with 1.8 ± 0.4% of the library being converted to a ligation product by 90 min (Fig. 3*A*). A similar rate of ligation was observed using a fluorophore-labeled primer in place of the RNA library, indicating that this corresponds to the background nonenzymatic rate of ligation (*SI Appendix*, Fig. S3). In contrast, the ligation product was observed by 5 min for all 10 sequences RS1–RS10 (Fig. 3*B* and *SI Appendix*, Fig. S4). We observed a fast rate of catalyzed ligation for the first 30 min of the time course, but from 30 to 90 min, the reaction rate decreased over time (Fig. 3*C*). Competing side reactions such as hydrolysis of the 2AI-activated ligator, which would inactivate the reaction complex, were not observed to be catalyzed by RS1 (*SI Appendix*, Fig. S5), suggesting that the plateau in ligation yield is likely due to partial misfolding of the ribozymes. Therefore, we nonlinearly fit the data for the ribozyme-catalyzed reactions to a first-order rate equation that accounts for unreactive complexes in order to obtain rate constants. We determined that the rate constants for ligation catalyzed by RS1–3 range from 2.4 to 6.2 h^−1^, while the nonenzymatic rate under these conditions is 0.014 h^−1^, indicating rate enhancement by a factor of 170–440.

### Catalyzed Ligation to the 3′ End of the Library.

Since a previous ribozyme selection using an imidazolide substrate unintentionally enriched for 5′,5′ ligases instead of 3′,5′ ligases (29), it was important to characterize the ligation junctions generated by our newly identified functional sequences RS1–RS10. The gel shift observed in our ligation assays (Fig. 3*B*) does not by itself discriminate between attachment of the 2AI-activated ligator to the 3′ end, the 5′ end, or an internal branch site. We reasoned that the desired 3′,5′ ligase activity should depend upon the presence of the template strand, 2AI activation of the ligator strand, and the 3′ terminal primer region of the RNA construct. For example, template-independent ligase activity was observed in the previous selection that identified the 5′,5′ ligase ribozymes (29). In addition, deletion of the primer region from the candidate sequences should abolish activity of a template-directed ligase but would not necessarily affect ligation at the 5′ end of the sequence or at an internal site.

**Fig. 4.**
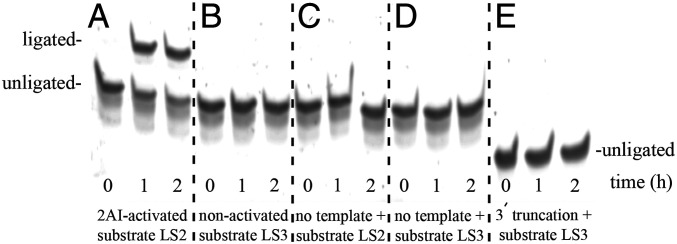
Ribozyme RS1 catalysis of ligation requires 2AI-activated substrate, the RNA template, and the primer at the 3′ end of the ribozyme sequence. All displayed samples are contained on a single gel, with different experiments separated by dashed lines. (*A*) Catalyzed ligation is observed by 1- to 2-h incubation of the RS1 ribozyme with template and 2AI-activated substrate LS2. (*B*) Replacing the 2AI-activated substrate with its unactivated counterpart LS3 containing a 5′ phosphate results in no detectable ligation by 2 h. (*C*) Removing the template from the assay eliminates ligase activity. (*D*) Ligase activity is also abolished in reactions containing the unactivated LS3 substrate and no template strand. (*E*) Truncating the 3′ end of RS1, which contains the primer region, prevents ligation with the 2AI-activated ligator.

To determine the template and activation requirements of the full-length sequences RS1–RS10, we tested each sequence in ligation reactions either with or without the template strand, and with either a 2AI-activated ligator or an unactivated ligator with a 5′ phosphate. We also prepared truncated RS1–RS10 RNA constructs lacking the 3′ terminal primer region and U6 linker and tested these in a ligation assay containing both the template strand and 2AI-activated ligator. Consistent with ligation occurring at the 3′ end of a primer, these three experimental conditions abolished ligase activity for all 10 ribozymes (Fig. 4). Furthermore, 1- and 2-nt deletions at the 3′ end of the primer or a 2-nt deletion at the 5′ end of the ligator both drastically reduced ligation by RS1 (*SI Appendix*, Fig. S6). Disrupting Watson–Crick base pairing at the ligation junction also reduced the ligase activity of RS1, especially with pyrimidine substitutions at the 3′ end of the primer (*SI Appendix*, Fig. S7).

**Fig. 5.**
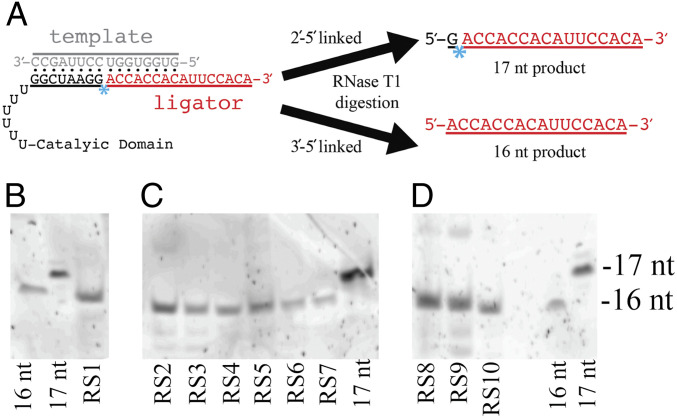
RS1–10 catalyze 3′-5′ phosphodiester bond formation. (*A*) Schematic for testing regiospecificity. This assay utilizes a modified ligator sequence with two G to A substitutions, so that the ligator will not be cut by RNase T1. If a 3′-5′ bond is created by the ligase, RNase T1 digestion generates a 16-nt product. Since RNase T1 cannot cleave a 2′-5′ bond, a 2′-5′ linkage in the product would survive T1 digestion and the 3′-5′ bond immediately upstream would be cleaved to generate a 17-nt product. Blue asterisk indicates location of phosphodiester bond being assayed. (*B*) PAGE of synthetic 16-nt and 17-nt standards beside the RNase T1 digestion product of RS1-catalyzed ligation. (*C*) Analysis of RS2–RS7 beside the 17-nt standard. (*D*) Analysis of RS8–10 beside the 16-nt and 17-nt standards.

Although these results indicate that ligation occurs in a template-directed manner at or near the primer region of the ribozyme, they cannot on their own distinguish the nature of linkage being formed. Therefore, we analyzed the ribozyme-catalyzed ligation products by RNase T1 digestion (Fig. 5), which cleaves 3′-5′ phosphodiester linkages, but not 2′-5′ linkages, that are 3′ relative to guanosine nucleotides (36). To perform this assay, the sequence of the 2AI-activated ligator was altered to replace all guanosine nucleotides with adenosine, and an appropriate template was used to maintain Watson–Crick base pairing throughout. These nucleotide changes make the entire ligator resistant to RNase T1 cleavage, but not the primer region of the ribozyme, which terminates in two guanosines. If the ligated products are 3′-5′ phosphodiester linked, then RNase T1 digestion will cleave the newly formed phosphodiester bond and generate a 16-nt product corresponding to the modified ligator strand with a 5′ hydroxyl group (Fig. 5*A*). Ligation products containing a 2′-5′ linkage will be resistant to RNase T1 digestion and result in a 17-nt product corresponding to the 16-nt ligator strand plus a single guanosine nucleotide at the 5′ terminus.

For all 10 ribozymes, our RNase T1 digestion assays indicate that the ligation reaction must generate a 3′-5′ phosphodiester linkage, which is most easily explained by ligation to the 3′ hydroxyl of the ribozyme construct (Fig. 5 *B*–*D*). Gel electrophoresis of the RNase T1 digest showed a product 16 nt in length, as determined by comigration with the 16-nt, but not the 17-nt, synthetic standards. In triplicate experiments, these gels did not show an observable band corresponding to the 17-nt product containing a 2′-5′ linkage. However, we cannot entirely exclude the possibility that a small fraction of the ligation products, below our limit of detection, contains a 2′-5′ linkage. Based upon the observed intensities of the gel image, we estimate that our sensitivity for detecting a 2′-5′ linked product to be 2.5–5% of the total product (*SI Appendix*, Fig. S8).

### Sequence Requirements for Ribozyme-Catalyzed Imidazolide Ligation.

In the ligation reactions described above, a catalytic RNA construct of 95 nt reacts with a ligator strand of 16 nt to generate a 111-nt product. We considered two regions to be potentially dispensable for this reaction: the first 25 nt at the 5′ end of the functional RNA sequence and the terminal 8 nt at the 3′ end of the ligator strand. The 25 nt at the 5′ end of the functional RNA sequences were included for PCR amplification during in vitro selection but may also play an important role in the folding and catalytic activity of these newly identified ribozymes. Secondary structure prediction suggested that some of the sequences (RS2, RS3, RS4, RS6, RS7, RS9) do not fold into the predesigned stem-loop structure (Fig. 1) but instead adopt alternative structures due to extensive pairing of the intended random loop with the 5′ end used for PCR amplification (37). Likewise, the 8-nt overhang at the 3′ end of the ligator strand was included during the selection to promote reaction quenching with a cDNA strand but was not designed to play a role in the catalytic function of the ribozymes.

**Fig. 6.**
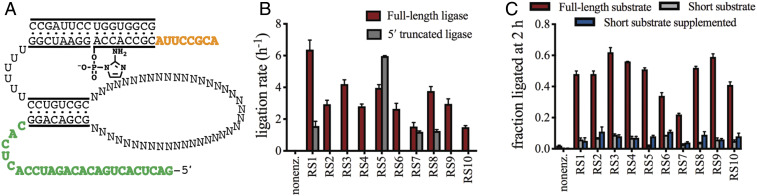
Effect on ligase activity of deleting the 5′ constant region of the RNA library and the 3′ overhang sequence of the ligator. (*A*) Diagram of the selection library highlighting the 5′ constant region (green) used for PCR amplification and the 3′ overhang sequence of the ligator (orange) included for reaction quenching. (*B*) Comparison of the ligation rate for the full-length 95-nt ribozyme and the 5′ truncated 70-nt RNA construct being tested. Rates for RS2, RS3, RS4, RS6, RS9, and RS10 are so low as to coincide with the *x* axis. (*C*) Fraction of the full-length ribozymes RS1–RS10 after 2-h incubation with the full-length 16-nt ligator LS2, the 8-nt truncated ligator LS4, and the 8-nt truncated ligator LS4 supplemented by OH1, which corresponds to the overhang sequence. Due to the observed plateau in ligation extents for all ribozymes under these conditions, the fraction ligated product at 2 h is an approximate measure of the catalyzed reaction yield.

To test the requirement for these two sequence regions, we prepared truncated RNA constructs for RS1–RS10 that lacked the first 25 nt of the 5′ end and a 2AI-activated 8-nt ligator strand lacking the overhang sequence (Fig. 6). Both full-length and 5′ truncated functional RNAs were incubated with either the 16-nt full-length or 8-nt shortened ligator strand, and reaction progress was monitored by gel electrophoresis. Of the 10 ligase sequences, the truncated versions of four sequences, RS1, RS5, RS7, and RS8, were active with catalytic rates comparable to their full-length counterparts (Fig. 6*B*). Eliminating the 25-nt sequence at the 5′ end of the ribozyme abolished activity for the other six ribozymes, possibly due to misfolding of the truncated sequences. These results are consistent with the secondary structure prediction, with the exception of RS7 and RS10. This differential response to 5′ truncations suggests distinct global folds for the selected ligase ribozymes, consistent with the lack of sequence similarity among most of the sequences (*SI Appendix*, Fig. S2).

The 3′ truncation of the 16-nt ligator resulted in a significant decrease in ligation efficiency by all 10 ribozymes (Fig. 6), with a maximum of 9% ligation after 2 h, only a modest increase from background ligation of 2% after 2 h. On careful examination of the enriched sequences, we found that the majority contained a region complementary to the ligator overhang, including RS1 (*SI Appendix*, Fig. S2). Ligation reactions containing the short ligator and a second 8-nt strand consisting of the overhang sequence only modestly increased the observed ligation yield for RS2, RS5, RS6, RS8, and RS10 and had no effect for the other ribozymes (Fig. 6). Additional ligation experiments using longer RNA template sequences that sequester the overhang further support the hypothesis of a long-range interaction between the ligator overhang and the catalytic RNA stem loop (*SI Appendix*, Fig. S9).

These experiments argue that the overhang plays a role in assembling the ribozyme–substrate complex into its catalytically competent configuration rather than only contributing to proper ribozyme folding. Potential interactions between the catalytic domain of the ribozyme and the 3′ overhang of the ligator may stabilize the catalytically competent fold of the ribozyme or help position the ligase at the imidazolide ligation junction. We determined the secondary structure of the 5′ truncated form of RS1 using a SHAPE assay in conjunction with the folding algorithm of the RNAStructure program (38–40). The secondary structure contains two loops separated by a 7-bp helix, with the more distal loop containing a sequence complementary to the ligator overhang (*SI Appendix*, Fig. S10). This distal loop is therefore capable of forming essential long-range interactions with the substrate to assemble the catalytically relevant active site.

### Comparison of Ribozyme-Catalyzed and Nonenzymatic Ligation.

2AI-activated RNA substrates nonenzymatically form phosphodiester bonds through two distinct mechanisms. The predominant mechanism of nonenzymatic RNA polymerization involves the formation of a covalent imidazolium-bridged intermediate, which can be the rate-limiting step of primer extension (41, 42). The second, slower reaction mechanism does not involve a covalent intermediate, but instead proceeds through a classic S_N_2 substitution reaction, whereby nucleophilic attack of the 3′ hydroxyl on a 5′-phosphor-imidazolide generates a new phosphodiester bond. Nonenzymatic RNA ligation is believed to occur through the classic S_N_2 substitution reaction because the concentration of an imidazolium-bridged ligator intermediate would be too low, based upon the previously measured second-order rate constants of imidazolium-bridged intermediate formation (41). In addition, such an intermediate was not observed in a previous analysis of 2AI-activated ligators (42).

To determine whether the mechanism of ribozyme-catalyzed ligation involves the formation of an imidazolium-bridged intermediate, we tested whether free 2AI inhibits the ligase activity. Free 2AI rapidly reacts with the imidazolium-bridged intermediate, thereby inhibiting phosphodiester bond formation through this pathway (41). We observed that addition of free 2AI to the RS1 ribozyme-catalyzed ligation reaction did not inhibit the ligation yield at 2 h (*SI Appendix*, Fig. S11). In addition, ligation reactions containing substoichiometric levels of ligator relative to ribozyme are not consistent with the second-order kinetics predicted for formation of a covalent intermediate (*SI Appendix*, Fig. S11). These results suggest that ribozyme-catalyzed and nonenzymatic RNA ligation occur through the classic S_N_2 substitution reaction pathway. Since ligation of 5′-triphosphate–activated substrates is believed to also occur through a similar in-line attack mechanism (43), we tested whether RS1 could also ligate a 5′-triphosphate–activated substrate, but no product was observed (*SI Appendix*, Fig. S11). Together, these results indicate that the ribozyme RS1 specifically catalyzes ligation of 2AI-activated substrates and that the mechanism does not involve a covalent, imidazolium-bridged intermediate.

The compact A-form structure typical of RNA duplexes is critical for nonenzymatic RNA polymerization, and switching RNA templates or monomers to DNA versions lowers the rate and yield of nonenzymatic reactions (44, 45). We tested whether RS1–RS10 also required RNA templates and substrates for ligation by changing the template and ligator to DNA. For all 10 ribozymes, replacing both the template and ligator with DNA counterparts drastically reduced the yield of ligation observed at 2 h (*SI Appendix*, Fig. S12). RS3, RS7, and RS10 retained substantial activity when DNA was used in only the template strand, while RS2 and RS10 retained activity when only the ligator was composed of DNA. These results suggest that the reduction of ligase activity in these experiments cannot be entirely accounted for by helical structure, as DNA-RNA hybrids are typically A-form. Substituting an RNA substrate with DNA of the same sequence might also disrupt ribozyme–substrate interactions involving 2′ hydroxyl groups, as observed in certain self-cleaving ribozymes (46, 47)

### Effects of pH and Divalent Cations on Ribozyme Catalysis.

**Fig. 7.**
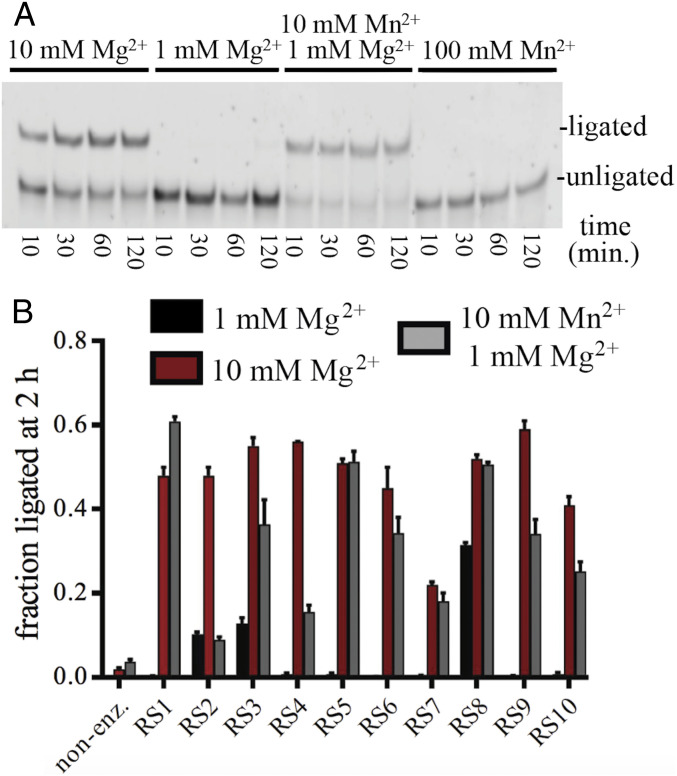
Ligase activity can be supplemented by Mn^2+^ under low Mg^2+^ concentrations. (*A*) PAGE of RS1 ligation reactions in the presence of 10 mM Mg^2+^, 1 mM Mg^2+^, 1 mM Mg^2+^ + 10 mM Mn^2+^, and 100 mM Mn^2+^. (*B*) Quantification of ligation for RS1–RS10 at 2-h reaction with 1 mM Mg^2+^, 10 mM Mg^2+^, and 10 mM Mn^2+^ with 1 mM Mg^2+^.

Nonenzymatic ligation can utilize a variety of divalent cations to catalyze phosphodiester bond formation (5), whereas the activity of functional RNA molecules can depend upon the presence of Mg^2+^ ions (48–50). Therefore, we investigated how the activity of the ligases depended upon the concentration and identity of the divalent cation. These sequences were selected in the presence of 5–20 mM Mg^2+^, but ligase activities were greatly diminished when the concentration of Mg^2+^ was lowered to 1 mM for all 10 ribozymes except RS8 (Fig. 7*A*). Supplementing 1 mM Mg^2+^ with 10 mM Mn^2+^ restored the ligase activity of most sequences, but Mn^2+^ on its own did not support ligase activity, even at 100 mM concentration (Fig. 7*A*). RS2 and RS4 did not greatly tolerate substitution of Mg^2+^ with Mn^2+^, suggesting that these ribozymes specifically interact with Mg^2+^ or that Mn^2+^ disrupts the function of these sequences. An interaction of the ribozyme RS9 with Mg^2+^ was also supported by Mg^2+^ titration studies comparing both ribozyme-catalyzed and nonenzymatic ligation reactions (*SI Appendix*, Fig. S13).

One of the potential roles of a catalytic divalent ion is to facilitate the deprotonation of the 3′ hydroxyl group on the primer, thereby accelerating nucleophilic attack on the 2AI-activated substrate. We measured ligation rates of RS1 at pH values between 6 and 11.5 to generate a pH-rate profile (*SI Appendix*, Fig. S14). From pH 6 to pH 9, ligation rate increases with an increase in pH and then decreases from pH 9 to pH 11.5. A similar trend has been previously observed in uncatalyzed ligation of triphosphate substrates (4). This pH-rate profile can be explained by an increase in rate due to enhanced deprotonation of the 3′ hydroxyl nucleophile from pH 6 to pH 9, followed by RNA denaturation at higher pH due to deprotonation of nucleobases. A plateau in the rate of ribozyme-catalyzed triphosphate ligation from pH 7.5–9 has also been previously observed, but rates at a pH above 9 were not reported in this study (51). Notably, the optimal pH for RS1 does not correspond to the p*K*_a_ of the 2AI leaving group, as might be expected if the reaction mechanism involved an imidazolium-bridged intermediate. In both nonenzymatic and ribozyme-catalyzed ligation of triphosphate substrates, the pH-rate profiles reveal a log-linear region with a slope equal to 1, suggesting that the rate-limiting step involves abstraction of a single proton (4, 51). We observed a log-linear region from pH 6–8, but our fit to this data resulted in a slope equal to 0.63. This deviation from the expected slope of 1 might be explained by the protonation state of the 2AI leaving group, whose p*K*_a_ is equal to 8. At lower pH, 2AI is protonated, making it a better leaving group, while at higher pH, 2AI is uncharged, making it a worse leaving group for the ligation reaction.

## Discussion

We have used in vitro selection, combined with the inherently reactive substrates of nonenzymatic RNA ligation, to demonstrate that template-directed ligation of a 2AI-activated oligonucleotide to form a phosphodiester linkage is a viable target reaction for ribozyme catalysis. This result shows that nonenzymatic and ribozyme-catalyzed RNA copying can be linked in models of the emergence of life. A previous report selecting for ribozymes that use an imidazolide substrate unintentionally enriched for 5′-5′ ligase activity, but our modified selection protocol successfully identified 3′-5′ ligases. Deep sequencing identified 64 independently derived sequences enriched through selection, and template-directed imidazolide ligase activity was observed for all 10 of the enriched sequences in the RNA pool we examined after eight rounds of selection, suggesting that ribozyme catalysts of this type of reaction may be rather common. Furthermore, 4 of 10 ribozymes retained ligase activity after removal of a 5′ constant region used for PCR amplification, reducing the length of the RNA sequence to 70 nt. This length is conceivably attainable through a combination of nonenzymatic polymerization and ligation reactions, as has been recently suggested for other RNA sequences (52, 53). We note that in vitro selection does not enrich for the smallest functional sequence and propose that even smaller ribozymes could be effective catalysts of the template-directed ligation of 2AI-activated substrates. Our isolation of multiple distinct small ribozymes that can ligate 2AI-activated substrates supports our view that early ribozymes emerging through nonenzymatic processes would have been able to catalyze phosphodiester bond formation using prebiotically plausible substrates.

Exactly how our newly isolated imidazolide ligases catalyze phosphodiester bond formation is currently unclear. Structural studies of nonenzymatic ligation suggest that the imidazole leaving group is disordered, thereby impeding in-line attack by the hydroxyl groups of the primer (26). Therefore, the catalytic mechanism of these newly identified imidazolide ligases could involve preorganization of the 2AI leaving group for in-line attack. The conformation of the 2AI group could be restricted through a variety of noncovalent interactions with the ribozymes, such as stacking or hydrogen bonding. Alternatively, the ribozymes could also stabilize the interaction of the Mg^2+^ or Mn^2+^ cation to enhance the nucleophilic attack of the primer on the activated ligator, similar to the mechanism of the class I ligase ribozyme (43). Whether all of the ligase ribozymes identified here operate through distinct or common mechanisms remains to be determined.

Although our selection strategy successfully identified imidazolide ligases, there remains a large gap between this catalytic activity and the ultimate goal of protein-free RNA copying. To this end, short ligase ribozymes could play an important role within a larger context of predominantly nonenzymatic polymerization reactions. For example, short precursor oligonucleotides formed through nonenzymatic template-directed polymerization of imidazolide monomers could then become substrates for a ligase ribozyme. Since nonenzymatic ligation of 2AI-activated oligonucleotides is much slower than polymerization of 2AI-activated monomers (12, 25), ligase ribozymes are particularly well suited for assembling precursor oligonucleotides into full-length functional sequences. Ribozymes could also have a role in both the polymerization and ligation of imidazolide substrates, especially A-U rich RNA sequences that are difficult to copy nonenzymatically. Continuing to evolve ribozymes with more general catalytic functions, and that capitalize upon the properties of chemical systems that mediate nonenzymatic RNA polymerization, will help to advance our understanding of primordial ribozyme-catalyzed RNA copying and its emergence during the origin of life.

## Methods

The sequences of DNA and RNA oligonucleotides used in this study are listed in the *SI Appendix*, Table S3. RNA pools and individual ligase ribozymes were prepared by Ava-II digestion of transcribed RNA or by transcription of DNA templates containing 5′ terminal 2′-O-methyl modifications to generate homogenous 3′ ends with free hydroxyls (33, 54). RNA ligator substrates were prepared as the 5′-phosphor-2-aminoimidazolide using standard activation procedures (12, 42). Detailed experimental procedures for the synthesis of RNA oligonucleotides, in vitro selection, high-throughput sequencing, ligation assays, and determination of regiospecificity are provided in *SI Appendix*.

### Data Availability Statement.

All data discussed in the paper will be made available to readers.

## Supplementary Material

Supplementary File

## Change History

September 29, 2021: The SI Appendix has been updated to coincide with a formal Correction.
