# Supplementary material for: In vitro selection of ribozyme ligases that use prebiotically plausible 2-aminoimidazole–activated substrates
Source: Proc Natl Acad Sci U S A. 2020 Mar 2;117(11):5741–8. doi: 10.1073/pnas.1914367117 (PMC7084097; doi:10.1073/pnas.1914367117)
Supplement: Supplementary File [file pnas.1914367117.sapp.pdf]

Supplementary Information

for

In vitro selection of ribozyme ligases that use prebiotically plausible 2-aminoimidazole-activated substrates

Travis Walton[a,*], Saurja DasGupta[a,*], Daniel Duzdevich[a], Seung Soo Oh[b], and Jack W. Szostak[a,1]

[a] Howard Hughes Medical Institute, Department of Molecular Biology, and Center for Computational and Integrative Biology, Massachusetts General Hospital, Boston, Massachusetts 02114, United States

[b] Department of Materials Science and Engineering, Pohang University of Science and Technology (POSTECH), Pohang, Gyeongbuk 37673, South Korea

[1] To whom correspondence should be addressed. Email: szostak@molbio.mgh.harvard.edu

* These authors contributed equally.

This PDF file includes:

Materials and Methods
Figures S1 to S14
Tables S1 to S3
SI References

MATERIALS AND METHODS

Materials:

All chemical reagents were purchased from Millipore Sigma, unless otherwise specified. Biotin-16-aminoallyl-2′-deoxycytidine-5′-triphosphate was purchased from TriLink BioTechnologies, Inc. The hydrochloride salt of 1-ethyl-3-(3-dimethylaminopropyl) carbodiimide was purchased from Alfa Aesar. The hydrochloride salt of 2-aminoimidazole was purchased from Combi-Blocks, Inc. 2,2′-dipyridyl disulfide was purchased from Chem-Impex International.

All enzymes and molecular biology reagents were purchased from New England Biolabs unless otherwise specified. Acid-phenol chloroform and SYBR™ Gold Nucleic Acid Gel Stain were purchased from ThermoFisher Scientific. 100% ethanol was purchased from Decon Laboratories, Inc. QIAquick PCR purification kits were purchased from Qiagen. The Sequagel-UreaGel concentrate and diluent system for denaturing 10% and 20% polyacrylamide gels was purchased from National Diagnostics. A 0.5 M ethylenediaminetetraacetic acid (EDTA) stock solution was purchased from Molecular Biologicals International, Inc.

All nucleic acid oligonucleotides are listed in the Supplementary Table S1. All oligonucleotides were purchased from Integrated DNA Technologies, except for LS3, which was synthesized in-house using standard RNA phosphoramidites purchased from BioAutomation.

Preparation of 2AI-activated oligos:

The biotinylated, 2AI-activated ligator LS1 used in the selection was prepared from the RNA oligomer LS3, which was converted to the 5′-phosphorimidazolide LS2 using triphenyl phosphine and 2,2′-dipyridyl disulfide as previously described (1). A biotin-16-aminoallyl-2′-deoxycytidine nucleotide was added to the 3′ end of LS2 to generate LS1 in an enzymatic reaction containing 10 μM substrate LS2, 25 μM DNA template BT1, 200 μM biotin-16-aminoallyl-2′-deoxycytidine-5′-triphosphate, 100 units/mL of Therminator™ DNA polymerase, and 1x of the vendor-supplied ThermoPol® reaction buffer. The Therminator™ reaction was incubated at 95 °C for 2 min., 4 °C for 10 min., and 37 °C for 1 h. This reaction was subsequently diluted with an equal volume of 1 mM $CaCl_2$ and digested with 50 units/mL DNase I for 30 min. at 37 °C. Then, the mixture was extracted with phenol/chloroform, ethanol precipitated, and purified by reverse phase analytical HPLC using a gradient of 98% to 75% 20 mM TEAB (triethylamine bicarbonate, pH 8) versus acetonitrile over 40 min. using procedures described previously (1). Fractions containing LS1 were flash frozen and lyophilized before determining purity by LC-MS. To prepare the 2-AI activated ligators LS2 and LS4 used in ligation assays, the RNA oligomers LS3 and LS5 were activated by incubating with 0.2 M 1-ethyl-3-(3-dimethylaminopropyl)carbodiimide (HCl salt) and 0.6 M 2-aminoimidazole (HCl salt, pH adjusted to 6) for 1-2 h at room temperature. The reaction mixtures were desalted and purified by HPLC as above. A similar approach was used for the other ligators used in this study.

In vitro selection:

The double-stranded DNA (dsDNA) library template was prepared in a 480 μL reverse transcription reaction containing 2.4 nmol DNA oligo SL1 and 4.8 nmol DNA oligo DS1 using ProtoScript® II reverse transcriptase enzyme according to manufacturer recommendations. The dsDNA library was converted to the RNA

library using HiScribe™ T7 polymerase in a 2.4 mL reaction incubated for 3 h at 37 °C. The T7 reaction was then digested by DNase I, extracted with phenol chloroform, ethanol-precipitated, and purified by 10% PAGE. The PAGE-purified T7 product RNA was then digested using the restriction enzyme Ava-II, which cleaves DNA/RNA hybrid duplexes to generate defined 3′ ends on the RNA strand with free hydroxyls groups (2). Ava-II digests were incubated for 10 h at 37 °C and contained 3.3 μM PAGE-purified T7 product, 10 μM DNA template AD1, 1x buffer, and 5000 units/mL Ava-II enzyme. Digests were then treated with DNase I, extracted with phenol chloroform, ethanol precipitated, and PAGE purified.

To select for ligase activity, RNA pools were incubated at room temperature for 10–120 min. in reactions that contained 1 μM RNA library, 1.1 μM RNA template LT1, 1.2 μM ligator LS1, 250 mM NaCl, 50 mM Tris buffer pH 8, and 5–20 mM $MgCl_2$. Amounts of pool RNA, concentrations of $MgCl_2$, and incubation time for each round of selection are provided in Supplementary Table S2. Ligation reactions were quenched with an equal volume of 200 mM EDTA, 200 μM DNA oligo RQ1, and 20 μM DNA oligo RQ2, and then heated at 95 °C for 2 min. Ligated RNA strands were then affinity purified using 1 mg of MyOne™ Streptavidin C1 Dynabeads® per 100 pmol RNA library. Dynabeads® were washed twice with buffer A (2 M NaCl, 2 mM EDTA, 20 mM Tris pH 7.6, 0.2% Tween-20), once with buffer C (25 mM NaOH, 50 mM NaCl, 1 mM EDTA), twice more with buffer A, blocked for 1 h in buffer A containing 10 μg / mL tRNAs, and finally washed twice again with buffer A. Biotinylated RNA was immobilized by 30 min. of shaking the washed Dynabeads® and the quenched ligation reaction at final concentrations of 1 mg / mL Dynabeads®, 0.1 μM RNA library, and 1x buffer A. The magnetic beads were then washed twice with buffer A, twice with buffer B (8 M urea, 1 M NaCl, 1 mM EDTA, 10 mM Tris pH 7.6, 0.1% Tween-20) for 15 min., twice with buffer C for 5 min., and three more times with buffer A. The magnetic beads were then suspended in 95% formamide containing 10 mM EDTA and heated for 6 min. at 65 °C to release the captured RNA. This sample was then diluted with 4.5 × volume of 300 mM sodium acetate with 100 ng / μL glycogen and ethanol-precipitated.

For rounds 1–3, ethanol-precipitated RNA was reverse transcribed in a reaction containing ProtoScript® II reverse transcriptase and 10 μM DNA primer RT1 according to manufacturer recommendations. Reactions were incubated at 42 °C for 3 h, and then heat-denatured for 5 min. at 80 °C. The denatured reverse transcription reactions were then diluted 10 × in a PCR reaction containing Taq DNA polymerase, 0.5 μM DNA primer PCR1, and 0.5 μM DNA primer PCR2 using manufacturer recommendations. PCR reactions were first incubated at 95 °C for 2 min.; then underwent 16 cycles of 94 °C for 30 s, 60 °C for 1 min., and 72 °C for 1 min.; and finally, 72 °C for 10 min. For rounds 4–8, ethanol-precipitated RNA was reverse transcribed in reactions containing 10 μM DNA primer RT2. The reverse transcribed pool was then PCR amplified as described above for only 10 cycles, purified using a QIAquick spin column, and amplified another 8 cycles using PCR. These steps were undertaken to reduce PCR amplification by the reverse transcription primer RT2, which is contained in the heat-denatured reverse transcription reaction. QIAquick purified PCR reactions were then transcribed by T7 RNA polymerase, and the RNA digested by Ava-II as described above.

High throughput sequencing:

To prepare the selected pools for sequencing, adaptor sequences were introduced onto the dsDNA pools resulting from each round of selection using several rounds of PCR with different primer sets. First, a 100 μL PCR reaction containing 25 ng dsDNA PCR product with 0.5 μM DNA primer PS1 and 0.5 μM DNA primer PS2, was amplified by Taq DNA polymerase for 4 cycles as described above. The PCR product was purified using QIAquick PCR Purification Kit (Qiagen), and then further amplified for 7 cycles in a second PCR reaction containing 40 ng dsDNA, 5 μM DNA primer PS3, and 5 μM DNA primer PS4. The purified product of the second PCR reaction was amplified once again in a 50 μL reaction containing 1 unit of Q5® Hot Start High-Fidelity DNA Polymerase (New England Biolabs), 100 ng of ds DNA, 1 × Q5 buffer, 0.2 μM each of SR Primer for illumina® and Index Primer for illumina® (New England Biolabs), and 80 μM dNTPs. This third PCR reaction was incubated at 98 °C for 30 s.; then underwent 6 cycles of 98 °C for 10 s, 62 °C for 15 s, and 72 °C for 20 s; and finally, 72 °C for 2 min. The PCR product was purified using a QIAquick PCR Purification Kit and then by agarose gel purification. The DNA was cleaned of residual dye and concentrated using an Oligo Clean & Concentrator Kit (Zymo Research) and submitted for deep sequencing.

Sequencing reads were filtered and counted using an R script. Reads were filtered based upon the presence of the predefined stem sequences and the quality ($Q \geq 20$) of the sequence corresponding to the loop region of the RNA library. The loop region of each read was then counted to provide sequence abundance for each round of the selection. Loop sequences were then clustered by thresholding the score of global pairwise alignment using the Biostrings package.

Ligation assays:

Ligation reactions contained 1 μM of the RNA pool or individual construct, 1.5 μM RNA template LT1, 2 μM ligator strand LS1 or LS2, 250 mM NaCl, 10 mM $MgCl_2$, and 100 mM Tris pH 8. Reaction aliquots of 1.4 μL were quenched in 6 μL kill buffer (8 M urea, 100 mM Tris-Cl, 100 mM boric acid, 75 mM EDTA) and stored frozen on dry ice until analysis by 10% PAGE. After running samples for 35 min. at 30 W, gels were stained using SYBR™ Gold and imaged on an Amersham Typhoon RGB instrument (GE Healthcare). RNA bands were quantified with IQTL 8.1 software, and then normalized relative to sequence length. Kinetic analysis was performed by nonlinearly fitting the data using Prism7 software to the modified first order rate equation $y = A(1 - e^{-kx})$, where A represents the fraction of active complex, *k* is the first order rate constant, x is time, and y is the fraction of ligated product.

Individual ribozymes and truncated constructs were prepared by *in vitro* transcription of DNA template containing 5′ terminal 2′-O-methyl modifications to reduce transcriptional heterogeneity at the 3' end of the synthesized RNA (3). Modified DNA templates were generated by PCR amplification using primers unique for each sequence and truncation product. Primers for generating 5′ truncations (FT1–10), 3′ truncations (TT1–TT10), and full-length constructs (ME1–ME10) using this method are included in Supplementary Table S3. One and two nucleotide 3′ truncations of RS1 (Fig. S6) were prepared using the primers ME1-1 and ME1-2. RS1 constructs with different 3′ terminal nucleotides (Fig. S7) were prepared with ME1-A, ME1-C, and ME1-U. Double-stranded DNA

products were then transcribed by T7 RNA Polymerase (NEB) in a reaction buffer containing 40 mM Tris-HCl (pH 8), 2 mM spermidine, 10 mM NaCl, 25 mM MgCl2, 10 mM Dithiothreitol (DTT), 30 U/mL RNase inhibitor murine (NEB), 2.5 U/mL thermostable inorganic pyrophosphatase (TIPPase) (NEB), 4 mM each NTP, and 30-60 pmol/mL DNA template, for 2 h at 37°C. The RNA was subsequently treated with DNase I, phenol-chloroform extracted, and PAGE-purified. Our initial examination of ligase activity in Figure 2 and S4 uses RNA prepared by Ava-II digestion as described above.

Determination of phosphodiester bond regiospecificity

Ligation reactions containing 10 pmol ribozyme, 10 pmol substrate oligo LS6, and 12 pmol of the DNA template LT2 were incubated for 2–6 h before DNase digestion to remove the template. Following phenol-chloroform extraction, unreacted LS6 substrate was removed by 3–4 washes on a centrifugal 30 kDa molecular weight filter (EMD Millipore). The resulting concentrate was denatured by incubation at 95 °C for 6 minutes in the presence of 6 M urea and 1 mM EDTA, and then digested by RNase T1 for 24 h at 55 °C. These digestions were then extracted by phenol-chloroform and analyzed by 20% PAGE. To determine the sensitivity of this assay, a calibration curve was generated using a synthetic 17 nt RNA oligo standard to quantify the limit of detection in terms of pmol input RNA (Fig. S14). We then quantified the 16 nt band intensities (corresponding to the RNase T1 digestion product of the 3′-5′ linked ligation product) for each of the reactions for RS1–RS10 and used these values to calculate pmol input RNA based upon the calibration curve. The sensitivity of our assay is then given by dividing the pmol limit of detection by the calculated pmol input RNA of the 16 nt digestion products. Multiplying this fraction by 100 gives the reported percentage value corresponding to the maximum amount of 2′-5′ linked ligation product possible in the sample without being detected in our assay.

Secondary structure determination by SHAPE

The secondary structure of RS1 was determined by adapting previously published RNA SHAPE protocols (4-6). The sequence used in the SHAPE experiments (5′-*GGCCUUCGGGCCAA***GGACAGCGGAAUGCUGC CAACCGUGCGGGCUAAUUGGCAGACUGAGCUCGCUGUCCUUUUUGGCUAAGG***UCGAUCCGGU UCGCCGGAUCCAAAUCGGGCUUCGGUCCGGUUC*-3′) consists of the 5′ truncated form of RS1 (bold letters) flanked by 5′ and 3′ SHAPE cassettes (italicized letters). The italicized, underlined letters correspond to the reverse transcription primer binding site. 100 pmol of this RNA sequence was folded under ligation conditions: 100 mM Tris-HCl (pH 8), 250 mM NaCl, 10 mM $MgCl_2$ and divided into two tubes for SHAPE modification and control reactions. 5 µl 100 mM 1M7 (in DMSO) was used per modification reaction. Following modification, both +1M7 and -1M7 RNA were reverse transcribed using 40 pmol 5′ FAM-labeled primer (5′-FAM- GAACCGGACCGAAGC CCG-3′). Sequencing lanes were generated by incorporating ddNTPs into reverse transcription reactions with 30 pmol unmodified RNA and 25 pmol FAM-labeled primer. The quenched reactions were then analyzed by 10% denaturing PAGE as described above. Normalized SHAPE reactivity was calculated by first excluding the most reactive nucleotide position and then dividing reactivities at each position by the average of the top 10% most

reactive positions (4 most reactive positions in this case). These normalized SHAPE reactivities were used to constrain secondary structure prediction in the RNAStructure program (4).

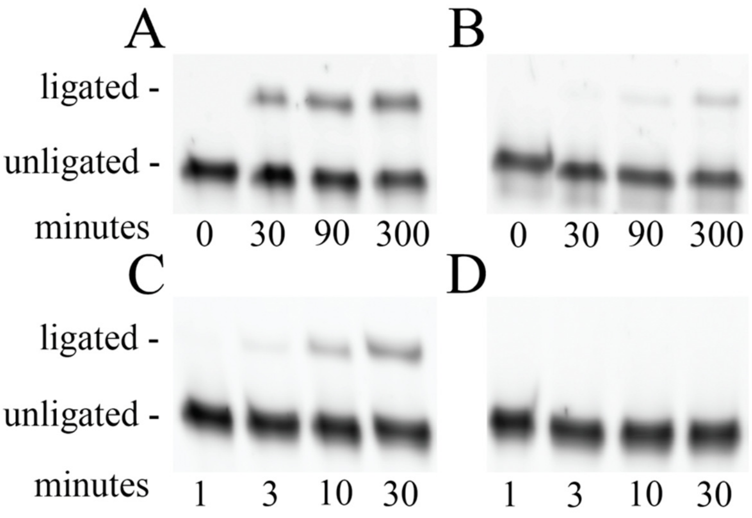

Figure S1. Observation of template-directed ligase activity in RNA pools after 6 rounds of selection. Time-course ligation reactions containing 1.1 μM RNA template LT1, 1.2 μM activated RNA ligator LS2, 20 mM $MgCl_2$, 250 mM NaCl, 1 M Tris pH 8, and either (A) 1 μM of the RNA pool after 6 rounds of selection, or (B) 1 μM of the initial naïve RNA pool were analyzed by 10% PAGE. (C) Stained gel images of 10 mM $MgCl_2$ ligation reactions containing the RNA pool after 7 rounds of selection with the template strand included, and (D) with the template strand omitted.

```
Cluster #                    Loop sequence

33------------------CGACAGCGCTCACCGTTGAAAGAGTTGCCAATGCAGAATA--------
29--------------GAACGCACAAACCTAGTGTCGGAATATTGTGCACTAAGCA------------
26--------------GAAACTGTTCACCTGGTGCTGGAAGGAACAGCCTAAGCGT------------
4 -------------GAACCCTTATCACAGTCGTGCGGATTTGTAAGCCTAAGCG-------------
32-------------GAACAGAGACTTAAATCGTGCTGGAATATCTCCCTAAGCG-------------
39-------------GAACCCGGAGGCAAATTGTGCGGACTCAACGAACTAAGCT-------------
13---------------------GACGGACGGTCGCGGTAACCTATGACCATGAGACGGAACA-----
60----------------------CCAAACGGGTTCGAAAAAACCACGCGGATTGGTATGACTC----
62------------------------ACAATCGTCGCTAAAAAACATGCGTGCCGGAATGTCGCAT--
53-----------TGATTGGGTTGGAGATACGCGGATGTATGATCTGCCGATC---------------
20--------------------------CTCTGCGCTGAAACGAAGCTGGAATAGCGCTACACTCAGA
21--------------TCCCGTGGGCGACCTACGGCGGAATGCTCATACGCTCAGT------------
9 ----------------CTGGCAAACACAGCGCGCTGTGTGTTATGTGGGG-CGGTCT---------
1 ----------------GAATGCTGCCAACCGTGCGGGCTAATTGGCAGAC-TGAGCT---------
5 -----------------AGAGACCGTGAGCTTGCGGAATGTTAGCAGAAC-AGAACTG--------
40--------TAATCCGGCACCCGTGGACCTCGAGCAAAATGCGGCCCCC------------------
58---------TCTATTCGAGTTTAAACCTTGCCGCGGAAGCCAAAACGTG-----------------
15------TTAGAGAGCCACATGCGCTCTGTTTTGCGGATAAAATGTG--------------------
31-------ATGTGCTAACACTTCTATAACCTTTGCGGAATGAAACCCC-------------------
3 TTAGTGAAATTGGTGCCCAAGCAGAGAATTGGGATAAATC--------------------------
55-------ATGCACTAAGGTTTTCGTTCTTCGTGTGGAATCAACATCG-------------------
19------AATTACCTGCCCATGTGCTGAATGCAGCGAAATCATCGAA--------------------
42------GTACTAAGATACAAAACCACGTCCTTGCGGAATCAACATA--------------------
54------GGACTAAGATCATTCCCCTGTTTATTGCGGAATCAACATA--------------------
24------CGTACTACCAACCAAGCCCCATCCGTGCGGAATGAACATC--------------------
64------TGTACTACGACTAAATGCCTAATGCTGCGGAATCAACCTC--------------------
48--------------------------TCAGGTGCGTGGTCGGCAACCACAGCGGATGAGTCCTAAC
36------------TGAATAGTACAGAGGCAGCTGCGGTCTACGTCCCTGCGCG--------------
52-------------GCTGTATAATTGGTCACGTTCGGAATACCTCAACCGGCGG-------------
10-----------CCGGTCTTAAGCCCCTGCGTTGCGGAAATGCACGTTGCCC---------------
34-------------CTCTTCCCAGACCTGAAGTGCGGAATTCTGTAGAGTCCCA-------------
47-----------AGGAATACGGTTAGATCCCCTGCGTTATGTGCCAGCGCAT---------------
18------------CGCCGACTCTAACTTAACCCGCGGAATGTGAGCGTAAACA--------------
37------ACAGCGGAGCAAACACAACTGCAGGTCGGGAATCCTGCCA--------------------
17----------GTGACCAGAGCCCAATTCAATCGCGGAATTCTTACACTAA----------------
57---------------AGGCGCCCAACTGAAGCGCGGAATGAAAAGACCCTCTCGT-----------
30----ACCCATTGGTTGGGTGACGTGGCATGTGACGGAATTGGCA----------------------
28--TGTCGTTGAGATATACTGGACAGACAAGACGTGGGAACTG------------------------
25-TCAGGAGGTAGCCCCATTGGACAGACTAGACGCGGAACAG-------------------------
2 -CCTAGCTAGCGCTGACTAGGACAGATGAGCGGCGGAACCA-------------------------
7 --AAAAGTTTCGCTGAATTGGACAGACCACCGCGTGAAGTGG------------------------
61--------------AGTAAAGTTAATACTGACACGGAATCAGAGATTGCTAATT------------
35------------AGTCCGTCTAAACGGTTATCGCGCGGAAGCGAGAGTAATT--------------
16-----------TGATGAATCGGCATACGTGGGTCAGAGTCATAGTGCGACA---------------
11-------------TTGGTGTAGAGCGCCAACTGGACAGACCTTACGG-AAACGG------------
12--------------AAGCTCTCGCCAGCAAAAGAACAGACCGTCGAGGAAACGG------------
38-------------ATAACTGTCGCCGTTTAATGGACAGACCAGTGCGG-AACGG------------
```

```
51--------------CGGCTTTAGACCGCATCTGGACAGATCAACGCGGACTTGA------------
45--------------GTCAGTCGTGAAACAATCGTACAGACGTTACCGGAAACCG------------
43--------------AGTCATATTTACCAAGGAGGACAGACATACACGGAATCTG------------
63--------------GTTACCAAAACCGCAAAATGACAGACGTCACCGGAAGCCG------------
14--------------AGAGCCTAGTCGCTAACCGGACAGACCTACACGGAATAGG------------
8 --------------TCAGTCGGAGTACCAGAGCGATAGACGTCCCCGGAAGCCG------------
49--------------TCAGCCGGAACAATAGAATGACAGACGTACGCGGAATACG------------
56---------TCGTTCCACGCGGGATCCCTTTAGAGGGGCTACAAATGCC-----------------
27-------GGTGACATTAAGCGGAATGCCTTAAGAGG-CGTACAAATAA------------------
44---------TTCTTCCATGCGGTTTTCCTTAAGAGGAG-TACAAAACTAT----------------
6 -----------AGCCACTGCGGAAGACCTTAAGAGGTGTAATTGCTCACCC---------------
41----ACACAGTTGAGAGTGCGGAATTCCTTAAGAGGAGCCCCTT----------------------
46-----------CCAGAACACGTCAACCCTGCCGAGGCTAAGGACATAAAAC---------------
23-------TGTCGTAAACGGTGTACCCCAAGTGAAGGGGTTGTAAATC-------------------
59--------GTAGTCAATGCCCAACCAACTCTGGCGGAATGGTAACTTA------------------
22------------TCAGTGAGAATACAAGTGT-GGAGGCTAAGTGACTTAAACC-------------
50------------TCAGGCATAGTAACCCAGTCGGAGGCTAAGGACG-TAAACC-------------
```

Figure S2. Multiple sequence alignment from Clustal Omega of the 64 peak sequences from clusters comprising > 0.1% of the round 8 deep sequencing reads. Regions complementary to the overhang sequence of the 2AI-activated ligator are highlighted in yellow. Regions complementary to the 8 bp stem (nucleotides 74–81 of the library, Figure 1A of the main text) are highlighted in cyan. The relatively few regions with perfect complementarity to the 5′ constant region (nucleotides 1–25) are shown in magenta, and those complementary to the ligation junction are highlighted green. Other regions of sequence alignment are highlighted in gray. For reasons of clarity, only sequences with ≥ 6 nt of perfect complementarity or alignment are shown. Clustal Omega was run with dealigned input sequences, 5 combined iterations, 5 maximum guide tree iterations, and 5 maximum HMM iterations.

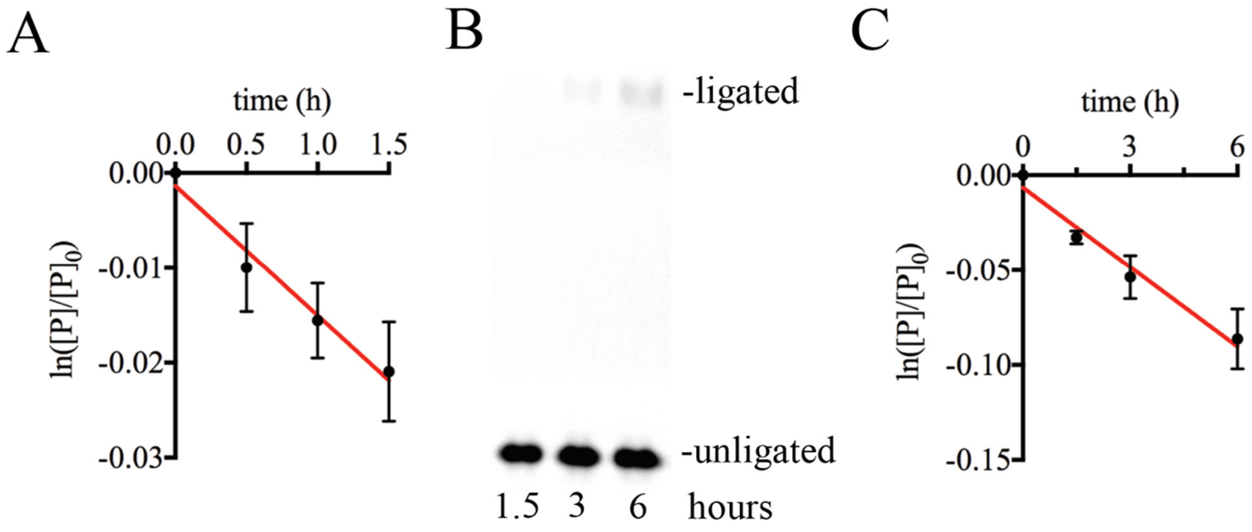

Figure S3. The ligation rate of the naive, unselected RNA pool does not differ from that of nonenzymatic ligation. (A) Quantification of ligation reaction of naïve RNA pool in a first order rate plot. The data presented here are also in Figure 3 of the main paper, but only the time points at 30, 60, and 90 min. are included in this plot because ligation product formation at earlier time points was too low for accurate quantification. The negative slope of the red line is equal to a first order rate constant of 0.014 ± 0.002 h^{-1}. (B) Nonenzymatic ligation reactions containing 1 μM labeled RNA primer LP1, 1.5 μM RNA template LT1, 2 μM activated RNA ligator LS1, 10 mM $MgCl_2$, 250 mM NaCl, and 1 M Tris pH 8 were analyzed by 20% PAGE over 6 h to favor observation of ligation product. (C) Quantification of nonenzymatic ligation rate in a first order rate plot. The negative slope of the red line is also equal to a first order rate constant of 0.014 ± 0.002 h^{-1}.

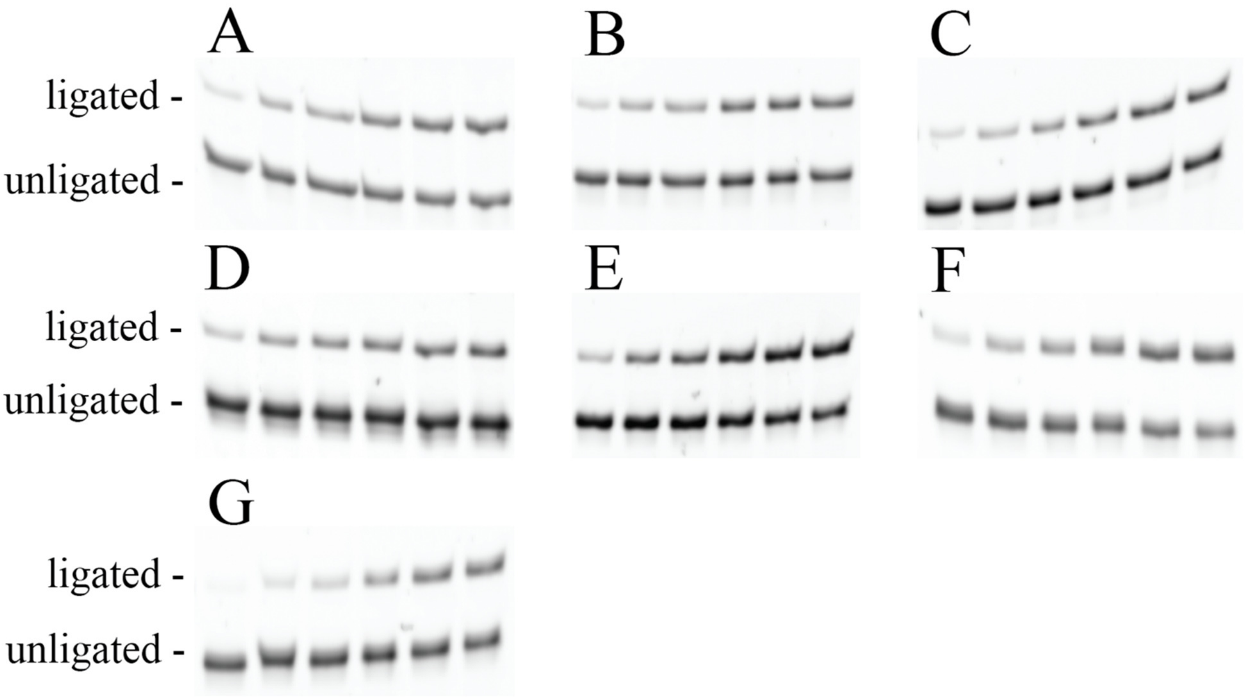

Figure S4. Observation of ligase activity within minutes for ribozymes RS4–RS10. Ligation reactions contained 1.5 μM RNA template LT1, 2 μM activated RNA ligator LS1, 10 mM $MgCl_2$, 250 mM NaCl, 1 M Tris pH 8, and 1 μM ribozyme construct (A) RS4, (B) RS5, (C) RS6, (D) RS7, (E) RS8, (F) RS9, or (G) RS10. Reactions aliquots were removed at 5, 10, 15, 30, 60, and 90 min. and analyzed by 10% PAGE with SYBR™ Gold staining.

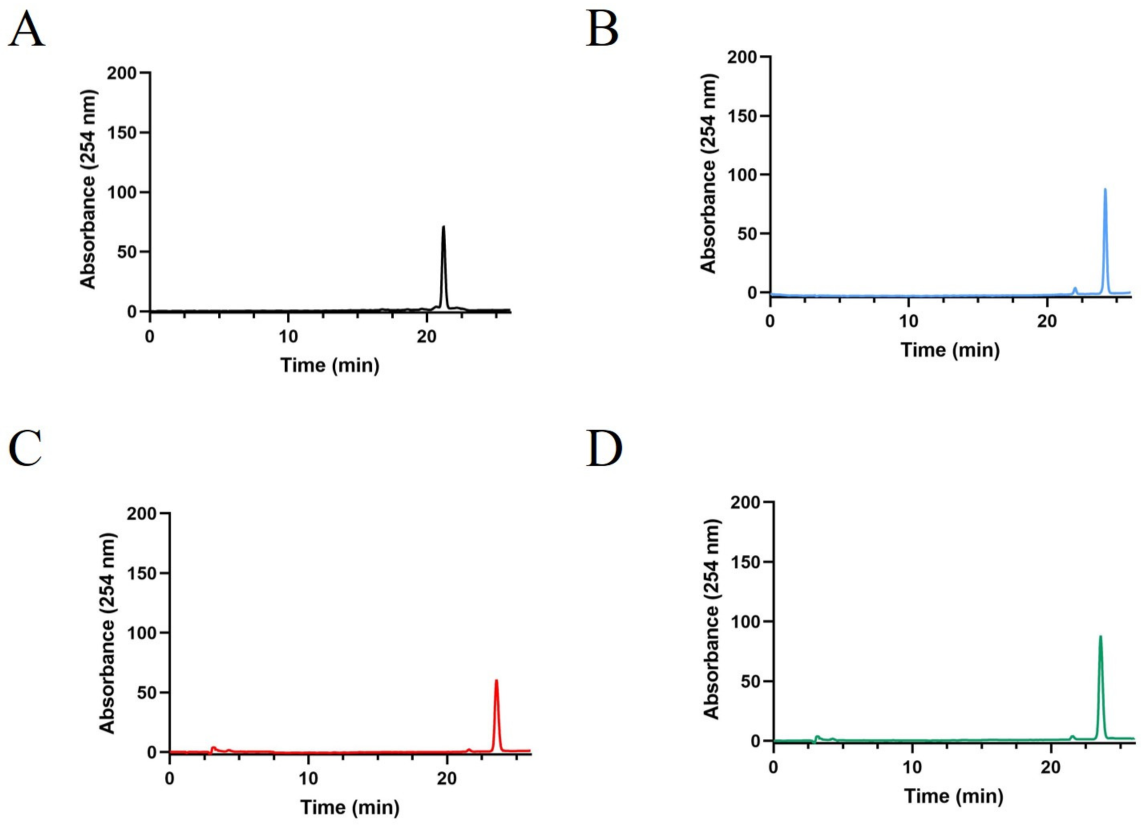

Figure S5. HPLC analysis reveals negligible hydrolysis of the 2AI-activated ligator in ribozyme ligation assays of RS1. (A) HPLC trace showing a peak at 21 min that corresponds to the 5′-monophosphorylated ligator LS3. (B) HPLC trace of 2AI-activated ligator LS2. The peak at 23 min. corresponds to LS2. The peak at 21 min. corresponds to a small amount of contaminating unactivated ligator LS3 in this preparation. (C) HPLC trace of the RNA ligator LS3 after 2 h incubation in reaction buffer without ribozyme. The fraction of hydrolyzed ligator is 2.4%. (D) Reaction containing 5′-phosphorimidazolide ligator with ribozyme. The fraction of hydrolyzed ligator is 2.9% This indicates that the ribozyme RS1 does not substantially catalyze hydrolysis of the 2AI-activated ligator.

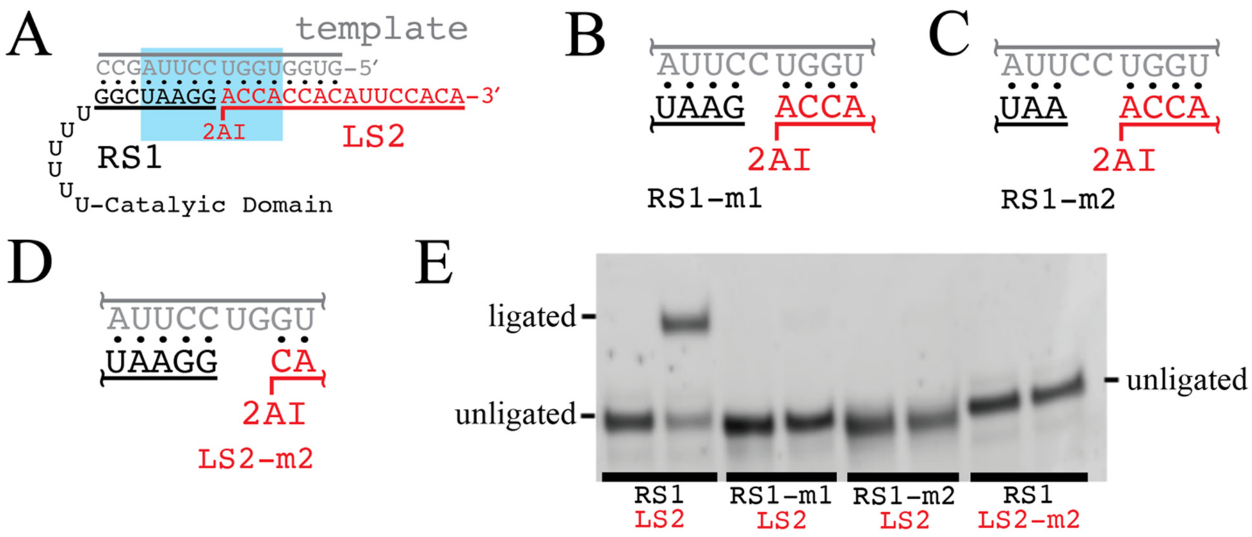

Figure S6. Gaps at the ligation junction disrupt the ligase activity of RS1. (A) Schematic of the reaction complex showing the ribozyme RS1 (black) and the 2AI-activated ligator LS2 (red) bound to a template (gray). The area surrounding the ligation junction is highlighted by a blue box. (B) Close up of the ligation junction for reactions containing RS1-ml and LS2, which is identical to the reaction complex in panel A, except that the 3′ end of RS1 is truncated by 1 nt. (C) The ligation junction for reactions containing RS1-m2, which is identical to the ribozyme RS1 except that its 3′ end is truncated by 2 nt. (D) The ligation junction of RS1 and LS2-m2. LS2-m2 is identical to LS2 except that its 5′ end is truncated by 2 nt. The 5′ terminal C nucleotide of LS2-m2 is activated with 2AI. (E) Analysis of ligation reactions containing full-length RS1 and LS2 constructs, as well as truncated variants, by PAGE. For each condition, the left lane is the starting material, and the right lane is after 2 h reaction. These results indicate that small perturbations to the predicted ligation junction decrease the formation of the ligation product, which is consistent with ligation to the 3′ end of RS1.

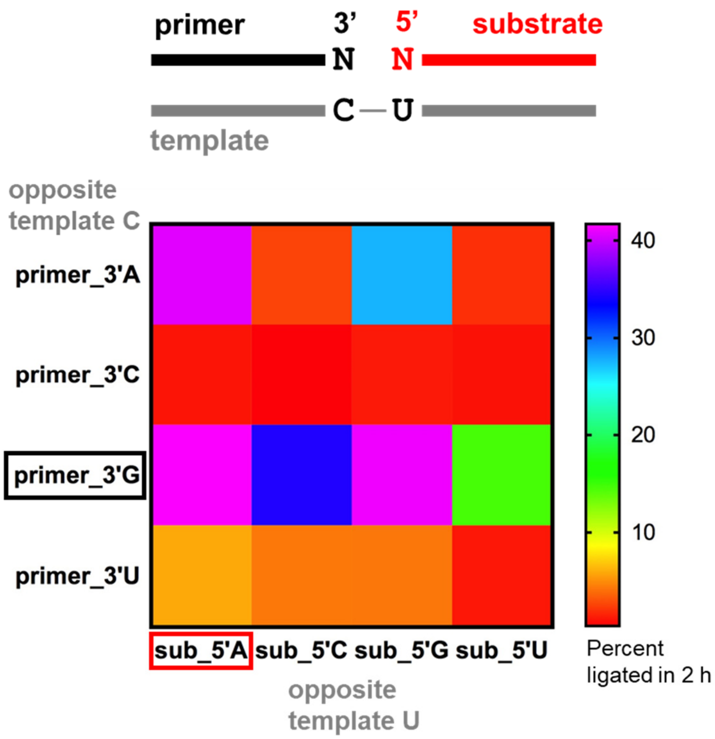

Figure S7. Base pair mismatches at the ligation junction attenuate ligation. The nucleotides flanking the ligation junction, the terminal 3′ nucleotide of RS1 and the 5′ terminal nucleotide of the ligator, were systematically changed and assayed in ligation reactions. Single-nucleotide variants of RS1 were prepared using ME1-A, ME1-C, and ME1-U, and used in combination with LS2, LS2-C, LS2-G, and LS2-U (Table S3). The template sequence was not changed in any of the reactions. The fraction of ligated product at 2 h was measured for the 16 different conditions and presented in a heat map. These results indicate that at least one intact base-pair is required for ligation; an AU or GU pair in the substrate-template duplex is sufficient to allow an AC mismatch in the primer-template duplex. Similarly, a GC pair in the primer-template duplex is sufficient to allow an CU (to some extent a UU) mismatch in the substrate-template duplex. Other mismatch combinations at the ligation junction do not support ligation.

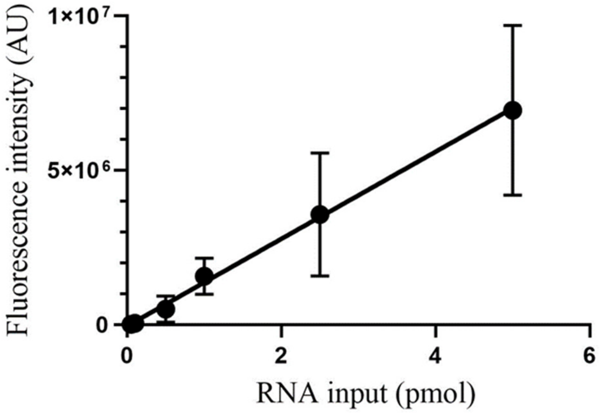

Figure S8. Calibration curve to determine sensitivity of RNase T1 digestion assay. 0.05–5 pmol of input RNA was analyzed by 20% PAGE followed by staining with SYBR gold. The input material is the synthetic 17 nt RNA standard seen in Fig. 5 of the main text. Fluorescence intensities of the input RNA were quantified in triplicate, and input RNA less than 0.1 pmol could not be distinguished above background noise. Based upon the observed fluorescence intensities of the 16 nt RNase T1 digestion products (Figure 5B–D of the main text), we calculate that the input RNA of RS1–10 reaction digests ranged from 2–4 pmol. This indicates that the regiospecificity assay should be able to detect amounts of 2′-5′ linked products greater than 2.5–5% of the total product.

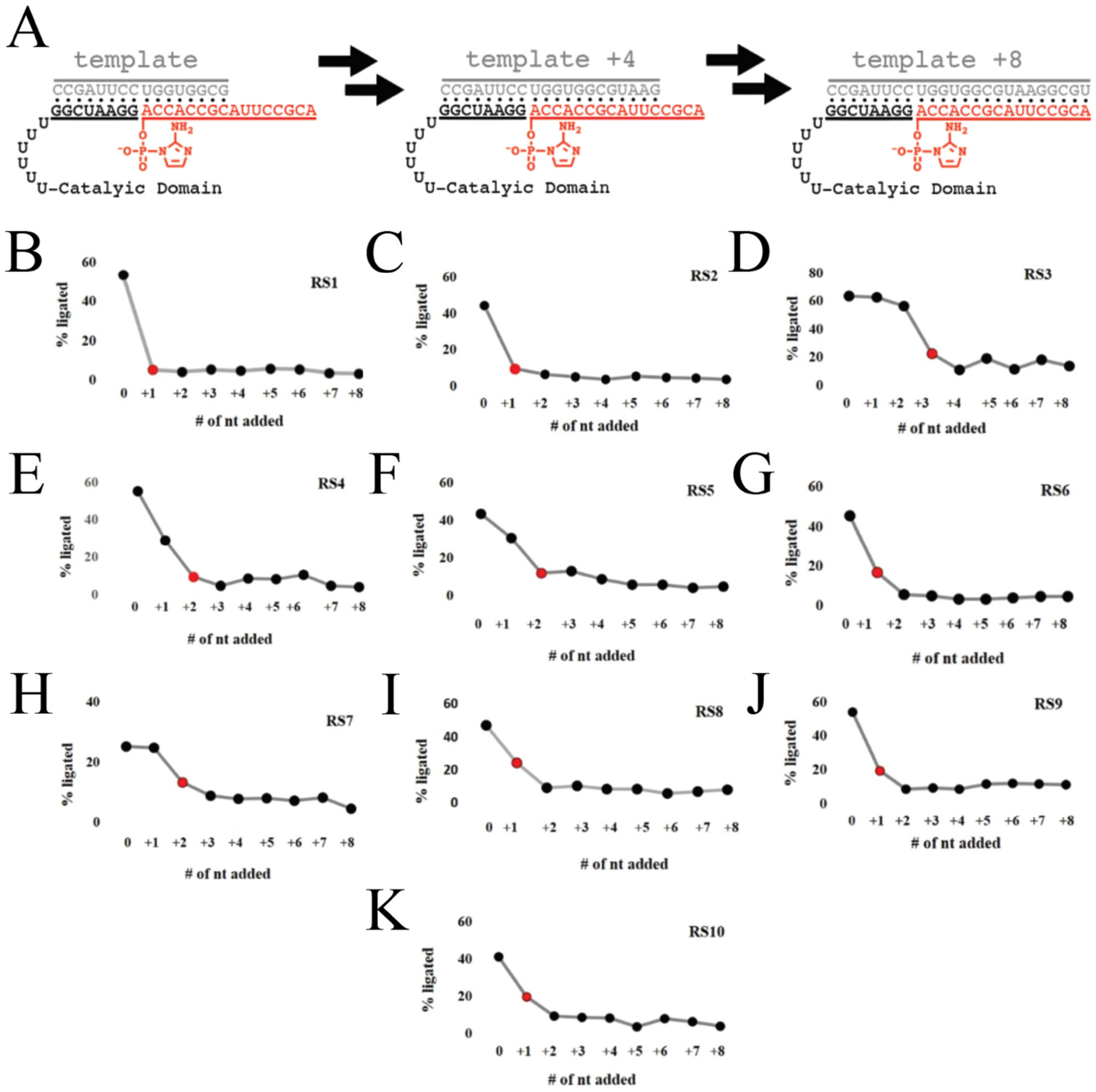

Figure S9. Sequential sequestering of the 3' substrate overhang results in decrease in ligation by ribozyme sequences RS1-10**.** (A) Extending the length of the template (gray) required for ligation sequesters the 3' overhang of the ligation substrate (red). (B–K) Ligation yields after 2h for ligases RS1–RS10 with templates strands of different sizes. The red dot indicates the length of template that reduces ligation yield by 50% relative to the standard 16 nt RNA template. Template sequences LT+1 through LT+8 can be found in Supplementary Table S3.

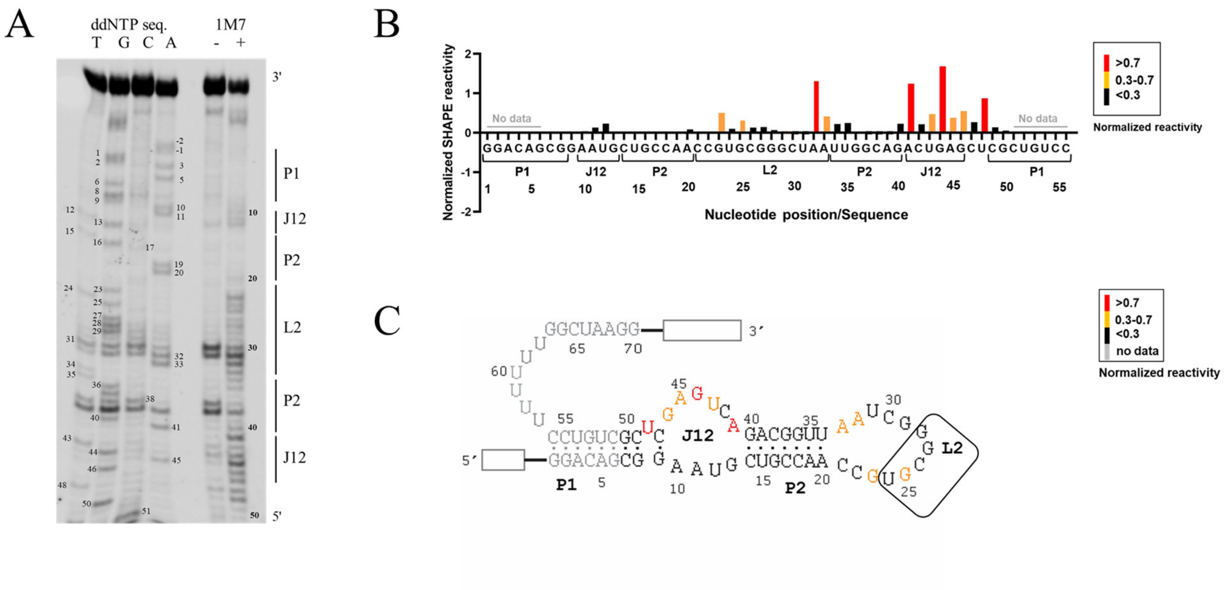

Figure S10. SHAPE analysis of the 5' truncated form of RS1. (A) Products of primer extension reactions with FAM-labeled primers separated on a 10% denaturing PAGE. T, G, C and A are sequencing reactions performed using ddATP, ddCTP, ddGTP, ddTTP terminating nucleotides, respectively. These marker lanes are 1 nucleotide longer than the corresponding 1M7 lanes. Nucleotide numbers next to the 1M7 lanes correspond to actual nucleotide positions. (–) and (+) 1M7 lanes represent reactions that differ in the presence of the SHAPE reagent. Regions such as base-paired stems (P1, P2), bulged junctions between two stems (J12) and loops (L2) are indicated next to the gel on the right. (B) Normalized SHAPE reactivities for each nucleotide position. High reactivity suggest flexibility within the RNA structure, while low reactivity suggests base-paired stems. A few nucleotide positions exhibited negative reactivity as is often observed in SHAPE experiments. These positions are assigned a reactivity value of 0. (C) Secondary structure of RS1 determined by the RNAStructure program (4) using reactivity constraints obtained from SHAPE experiments. 5′ and 3′ SHAPE cassettes are denoted by white rectangles. Notably, loop L2 contains a sequence of 5 nt (boxed, 5′-UGCGG-3′) that is complementary to the overhang sequence of the ligator strand (not shown). We propose that L2 interacts with the overhang sequence of the ligator to position the junction J12 at the ligation junction in order to catalyze phosphodiester bond formation.

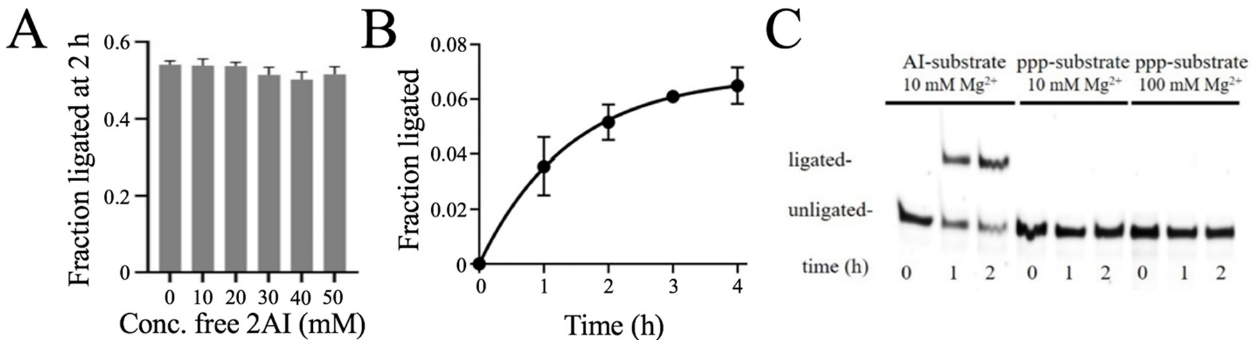

Figure S11. RS1 directly catalyzes ligation of the 2AI-activated substrate, but not a triphosphate-activated substrate. (A) Ligation yield at 2 h as a function of 0–50 mM free 2AI in the reaction buffer. Free 2AI inhibits nonenzymatic RNA polymerization by reducing the concentration of a reactive covalent intermediate, even at concentrations as low as 10 mM free 2-aminoimidazole (7). The yield of ligation product by RS1 after 2 h reaction is identical from 0 to 50 mM 2AI, suggesting that the ligation reaction does not proceed through a covalent intermediate. Higher concentrations of free 2-aminoimidazole slightly reduce the reaction yield, possibly through slight changes in reaction pH or other non-specific effects. (B) Kinetic plot of ligation progress over time in a reaction containing 1 µM RS4 and 0.1 µM 2AI-activated ligator. Nonlinear fitting of the data results in a first order rate constant 0.72 h^{-1} and a maximum calculated fractional yield of 0.07. In reactions containing 2 µM 2AI-activated ligator, the first order rate constant equals 2.76 h^{-1}. If the reaction mechanism involved a covalent imidazolium-bridged intermediate, then reaction rate should be second order with respect to the 2AI-activated ligator (7). However, decreasing the concentration of the ligator by a factor of 20 changed the calculated rate constant by a factor of < 4, while second-order kinetics predict that the change in ligator concentration should change the rate constant by a factor of 400. Therefore, the observed kinetics are not consistent with formation of an imidazolium-bridged intermediate. (C) RS1 does not significantly catalyze ligation of the 5′-triphosphate activated ligator LS8. PAGE of ligation reactions containing RS1 and the 2AI-activated or the corresponding 5′-triphosphate activated ligator at 0, 1, and 2 h. Ligation product is observed by 1 h when the 2AI-activated ligator is used in the presence of 10 mM Mg^{2+}, but no product is observed when the 5′-triphosphate activated ligator is used even in the presence of 100 mM Mg^{2+}.

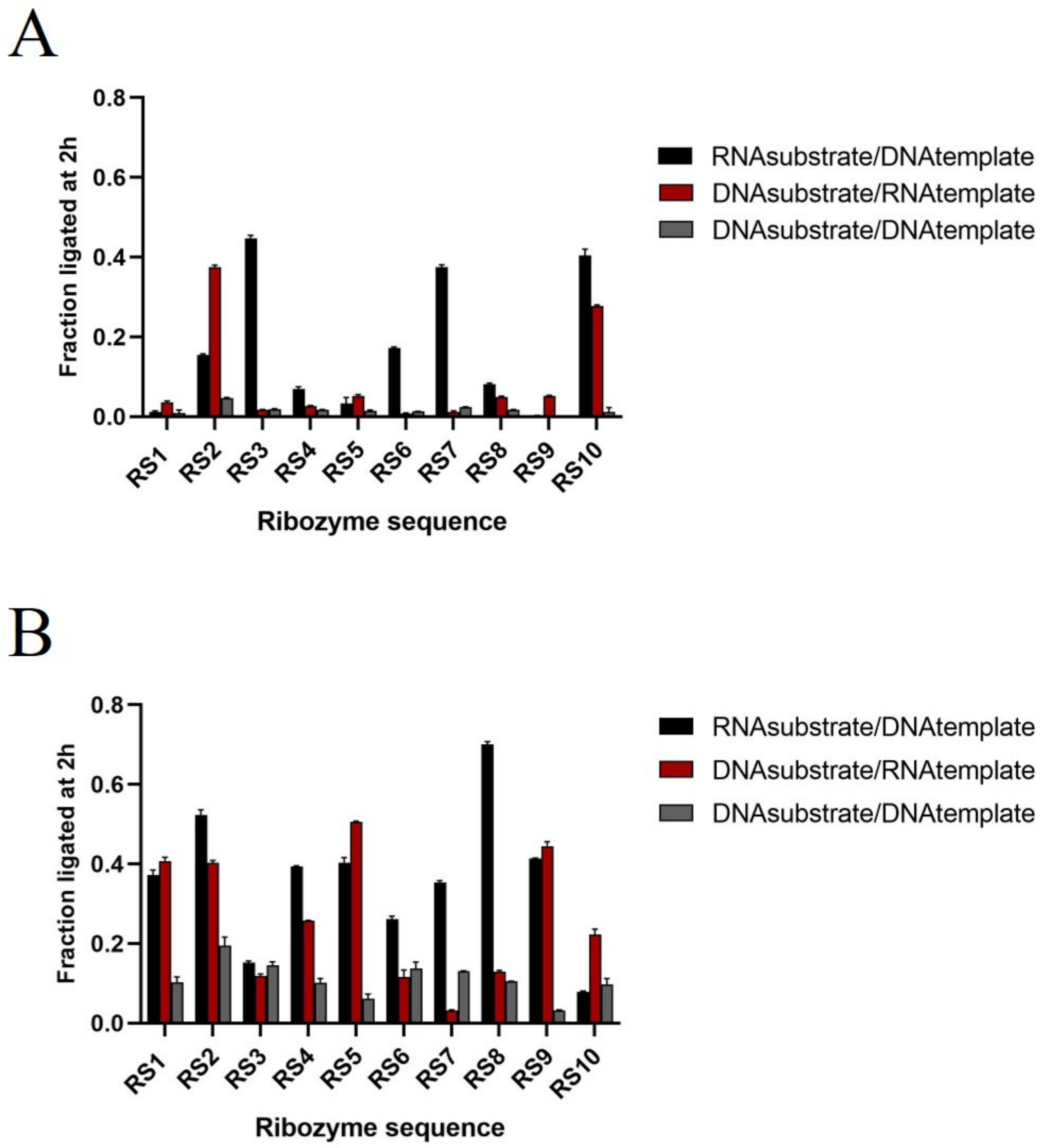

Figure S12. Ribozyme-catalyzed ligation of activated RNA substrate on DNA template and activated DNA substrate on RNA and DNA templates. The yield of ribozyme-catalyzed ligation reactions after 2 h were measured in the presence of (A) 10 mM Mg^{2+} and (B) 100 mM Mg^{2+} using the DNA ligator LS7 and DNA template LT2.

A B

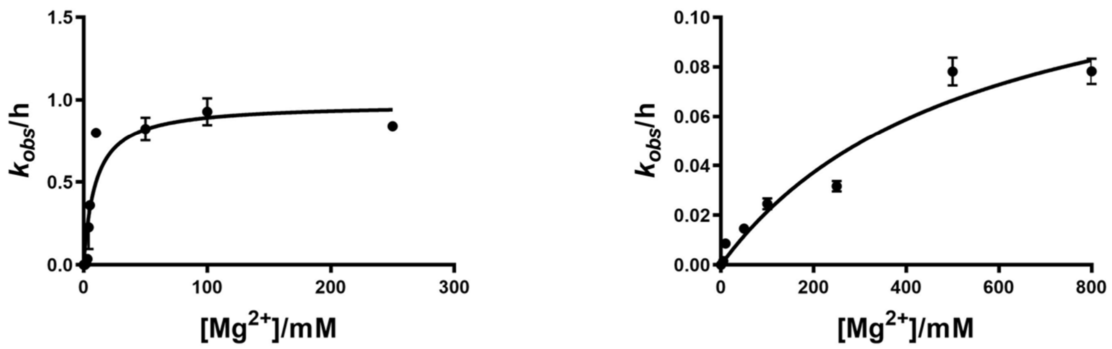

Figure S13. Ribozyme-catalyzed and nonenzymatic ligation rates as a function of Mg^{2+} concentration. (A) The rate of ligation by RS9 was measured in reactions containing 0–250 mM Mg^{2+}. RS9 was chosen for this analysis because minimal activity was observed at low Mg^{2+} concentrations (Fig. 7, main text). The rate of RS9-mediated ligation plateaus above 50 mM Mg^{2+}. The line is a fit to the Michaelis-Menten equation and provides a $[Mg^{2+}]_{1/2} = 9.5 \pm 3.5$ mM. (B) The rate of nonenzymatic ligation was measured in reactions containing 0–800 mM Mg^{2+}. In contrast to ligation by RS9, the rate of nonenzymatic ligation does not clearly plateau by even 250 mM Mg^{2+}. The fit to this data indicates a $[Mg^{2+}]_{1/2} = 546 \pm 269$ mM. The large difference in $[Mg^{2+}]_{1/2}$ between catalyzed and uncatalyzed reactions suggests a potential binding interaction of RS9 with Mg^{2+} that is important for catalysis.

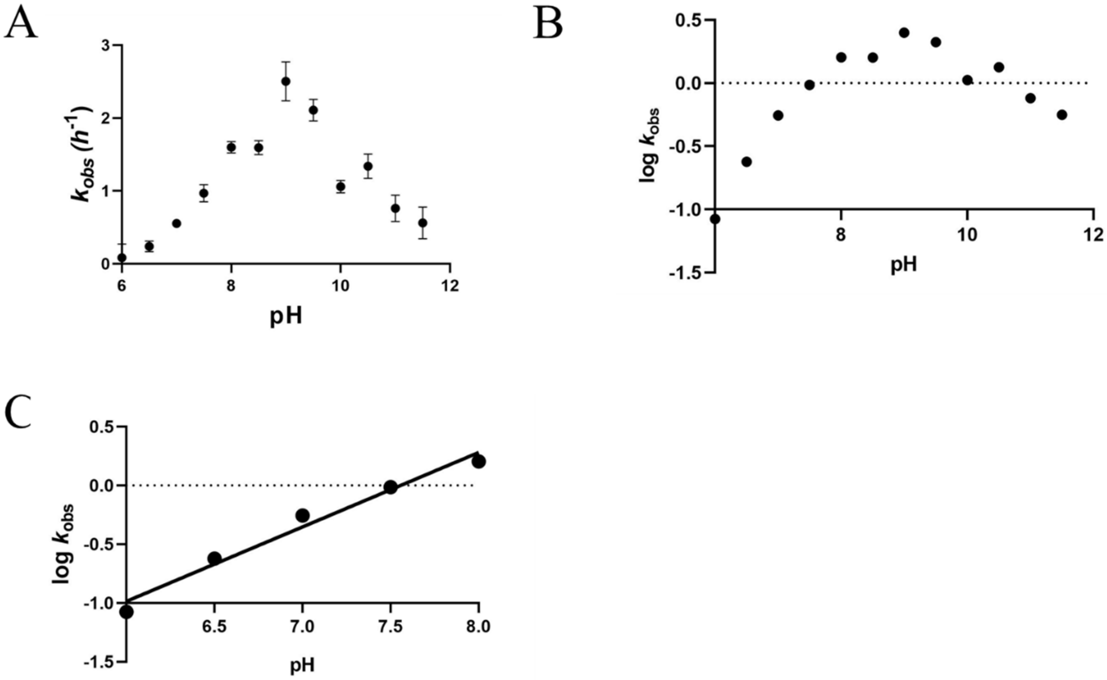

Figure S14. pH dependence on ligation rates of RS1. (A) Ligation rate increases from pH 6 to pH 9, and then decreases from pH 9 to pH 12. (B) Same data as in panel A, except that k_{obs} is log-transformed. (C) Subset of panel B showing that the increase in ligation rate is log-linear in the range pH 6-8 with a slope of 0.63.

round	scale (pmol)	time (min.)	RT primer	$MgCl_2$ (mM)
1	1100	120	RT1	20
2	320	60	RT1	20
3	100	30	RT1	20
4	100	20	RT2	20
5	50	30	RT2	20
6	50	10	RT2	20
7	50	10	RT2	10
8	50	10	RT2	5

Table S1. Parameters for each round of selection.

cluster rank	total reads of cluster	% abundance	loop sequence of most abundant member in cluster	# reads for most abundant	RS # (if applicable)
1	247909	39.655	GAATGCTGCCAACCGTGCGG GCTAATTGGCAGACTGAGCT	202551	RS1
2	69291	11.084	CCTAGCTAGCGCTGACTAGG ACAGATGAGCGGCGGAACCA	64295	RS2
3	36354	5.815	TTAGTGAAATTGGTGCCCAA GCAGAGAATTGGGATAAATC	32537	RS3
4	28844	4.614	GAACCCTTATCACAGTCGTG CGGATTTGTAAGCCTAAGCG	27524	RS5
5	22028	3.524	AGAGACCGTGAGCTTGCGGA ATGTTAGCAGAACAGAACTG	20466	RS10
6	13247	2.119	AGCCACTGCGGAAGACCTTA AGAGGTGTAATTGCTCACCC	12331	RS8
7	12902	2.064	AAAAGTTTCGCTGAATTGGA CAGACCACCGCGTGAAGTGG	11320	RS7
8	10803	1.728	TCAGTCGGAGTACCAGAGCG ATAGACGTCCCCGGAAGCCG	9192	RS4
9	10757	1.721	CTGGCAAACACAGCGCGCTG TGTGTTATGTGGGGCGGTCT	9773	RS6
10	7489	1.198	CCGGTCTTAAGCCCCTGCGTT GCGGAAATGCACGTTGCCC	6654	
11	6082	0.973	TTGGTGTAGAGCGCCAACTG GACAGACCTTACGGAAACGG	5470	
12	5755	0.921	AAGCTCTCGCCAGCAAAAGA ACAGACCGTCGAGGAAACGG	5141	RS9
13	5672	0.907	GACGGACGGTCGCGGTAACC TATGACCATGAGACGGAACA	5130	
14	5043	0.807	AGAGCCTAGTCGCTAACCGG ACAGACCTACACGGAATAGG	4557	
15	3859	0.617	TTAGAGAGCCACATGCGCTC TGTTTTGCGGATAAAATGTG	3425	
16	3416	0.546	TGATGAATCGGCATACGTGG GTCAGAGTCATAGTGCGACA	3117	
17	3286	0.526	GTGACCAGAGCCCAATTCAA TCGCGGAATTCTTACACTAA	2973	
18	3245	0.519	CGCCGACTCTAACTTAACCC GCGGAATGTGAGCGTAAACA	2925	
19	3208	0.513	AATTACCTGCCCATGTGCTG AATGCAGCGAAATCATCGAA	2953	
20	3092	0.495	CTCTGCGCTGAAACGAAGCT GGAATAGCGCTACACTCAGA	2858	
21	2887	0.462	TCCCGTGGGCGACCTACGGC GGAATGCTCATACGCTCAGT	2665	

22	2540	0.406	TCAGTGAGAATACAAGTGTG<br>GAGGCTAAGTGACTTAAACC	2214	
23	2533	0.405	TGTCGTAAACGGTGTACCCC<br>AAGTGAAGGGGTTGTAAATC	2300	
24	2449	0.392	CGTACTACCAACCAAGCCCC<br>ATCCGTGCGGAATGAACATC	2076	
25	2395	0.383	TCAGGAGGTAGCCCCATTGG<br>ACAGACTAGACGCGGAACAG	2166	
26	2330	0.373	GAAACTGTTCACCTGGTGCT<br>GGAAGGAACAGCCTAAGCGT	2028	
27	2165	0.346	GGTGACATTAAGCGGAATGC<br>CTTAAGAGGCGTACAAATAA	1705	
28	2106	0.337	TGTCGTTGAGATATACTGGA<br>CAGACAAGACGTGGGAACTG	1929	
29	1873	0.300	GAACGCACAAACCTAGTGTC<br>GGAATATTGTGCACTAAGCA	875	
30	1703	0.272	ACCCATTGGTTGGGTGACGT<br>GGCATGTGACGGAATTGGCA	1512	
31	1382	0.221	ATGTGCTAACACTTCTATAAC<br>CTTTGCGGAATGAAACCCC	993	
32	1326	0.212	GAACAGAGACTTAAATCGTG<br>CTGGAATATCTCCCTAAGCG	1244	
33	1222	0.195	CGACAGCGCTCACCGTTGAA<br>AGAGTTGCCAATGCAGAATA	1140	
34	1214	0.194	CTCTTCCCAGACCTGAAGTG<br>CGGAATTCTGTAGAGTCCCA	1101	
35	1212	0.194	AGTCCGTCTAAACGGTTATC<br>GCGCGGAAGCGAGAGTAATT	1101	
36	1140	0.182	TGAATAGTACAGAGGCAGCT<br>GCGGTCTACGTCCCTGCGCG	1058	
37	1094	0.175	ACAGCGGAGCAAACACAACT<br>GCAGGTCGGGAATCCTGCCA	1002	
38	1091	0.175	ATAACTGTCGCCGTTTAATG<br>GACAGACCAGTGCGGAACGG	967	
39	1090	0.174	GAACCCGGAGGCAAATTGTG<br>CGGACTCAACGAACTAAGCT	979	
40	1077	0.172	TAATCCGGCACCCGTGGACC<br>TCGAGCAAAATGCGGCCCCC	938	
41	1076	0.172	ACACAGTTGAGAGTGCGGAA<br>TTCCTTAAGAGGAGCCCCTT	978	
42	1029	0.165	GTACTAAGATACAAAACCAC<br>GTCCTTGCGGAATCAACATA	938	
43	979	0.157	AGTCATATTTACCAAGGAGG<br>ACAGACATACACGGAATCTG	872	

44	943	0.151	TTCTTCCATGCGGTTTTCCTT AAGAGGAGTACAAAACTAT	840	
45	932	0.149	GTCAGTCGTGAAACAATCGT ACAGACGTTACCGGAAACCG	852	
46	862	0.138	CCAGAACACGTCAACCCTGC CGAGGCTAAGGACATAAAAC	461	
47	854	0.137	AGGAATACGGTTAGATCCCC TGCGTTATGTGCCAGCGCAT	785	
48	849	0.136	TCAGGTGCGTGGTCGGCAAC CACAGCGGATGAGTCCTAAC	777	
49	803	0.128	TCAGCCGGAACAATAGAATG ACAGACGTACGCGGAATACG	731	
50	802	0.128	TCAGGCATAGTAACCCAGTC GGAGGCTAAGGACGTAAACC	664	
51	767	0.123	CGGCTTTAGACCGCATCTGG ACAGATCAACGCGGACTTGA	702	
52	763	0.122	GCTGTATAATTGGTCACGTTC GGAATACCTCAACCGGCGG	348	
53	762	0.122	TGATTGGGTTGGAGATACGC GGATGTATGATCTGCCGATC	647	
54	752	0.120	GGACTAAGATCATTCCCCTG TTTATTGCGGAATCAACATA	695	
55	742	0.119	ATGCACTAAGGTTTTCGTTCT TCGTGTGGAATCAACATCG	676	
56	741	0.119	TCGTTCCACGCGGGATCCCTT TAGAGGGGCTACAAATGCC	682	
57	715	0.114	AGGCGCCCAACTGAAGCGCG GAATGAAAAGACCCTCTCGT	656	
58	703	0.112	TCTATTCGAGTTTAAACCTTG CCGCGGAAGCCAAAACGTG	634	
59	688	0.110	GTAGTCAATGCCCAACCAAC TCTGGCGGAATGGTAACTTA	638	
60	667	0.107	CCAAACGGGTTCGAAAAAAC CACGCGGATTGGTATGACTC	607	
61	662	0.106	AGTAAAGTTAATACTGACAC GGAATCAGAGATTGCTAATT	612	
62	656	0.105	ACAATCGTCGCTAAAAAACA TGCGTGCCGGAATGTCGCAT	550	
63	653	0.104	GTTACCAAAACCGCAAAATG ACAGACGTCACCGGAAGCCG	567	
64	640	0.102	TGTACTACGACTAAATGCCT AATGCTGCGGAATCAACCTC	601	

Table S2. Sequence clusters with > 0.1% abundance identified in the round 8 pool.

Name	Type	Sequence
AD1	DNA	CGGTAGGTCCCTTAGCC
BT1	DNA	GTGCGGAATGCGGTGGTCC
DS1	DNA	TAATACGACTCACTATAGACTCACTGACACAGATCCACTCACGGACAGCG
FT1	DNA	TAATACGACTCACTATAGGGACAGCGGAATGCTGCCAA
FT2	DNA	TAATACGACTCACTATAGGGACAGCGCCTAGCTAGCG
FT3	DNA	TAATACGACTCACTATAGGGACAGCGTTAGTGAAATTGGTGCC
FT4	DNA	TAATACGACTCACTATAGGGACAGCGTCAGTCGGAGTAC
FT5	DNA	TAATACGACTCACTATAGGGACAGCGGAACCCTTATCACAG
FT6	DNA	TAATACGACTCACTATAGGGACAGCGCTGGCAAACAC
FT7	DNA	TAATACGACTCACTATAGGGACAGCGAAAAGTTTCGCTGAATTGG
FT8	DNA	TAATACGACTCACTATAGGGACAGCGAGCCACTGC
FT9	DNA	TAATACGACTCACTATAGGGACAGCGAAGC
FT10	DNA	TAATACGACTCACTATAGGGACAGCGAGAG
LP1	RNA	/5Cy3/rUrUrUrUrGrGrCrUrArArGrG
LS1	RNA/DNA	(5'-phosphoro-2AI)-rArCrCrArCrCrGrCrArUrUrCrCrGrCrA-(dC-biotin)
LS2	RNA	(5'-phosphoro-2AI)-rArCrCrArCrCrGrCrArUrUrCrCrGrCrA
LS3	RNA	(5'-monophosphate)-rArCrCrArCrCrGrCrArUrUrCrCrGrCrA
LS4	RNA	(5'-phosphoro-2AI)-rArCrCrArCrCrGrC
LS5	RNA	(5'-monophosphate)-rArCrCrArCrCrGrC
LS6	RNA	(5'-phosphoro-2AI)-rArCrCrArCrCrArCrArUrUrCrCrArCrA
LS7	DNA	(5'-phosphoro-2AI)-ACCACCGCATTCCGCA
LS8	RNA	(5'-triphosphate)-rArCrCrArCrCrGrCrArUrUrCrCrGrCrA
LS2-m1	RNA	(5'-phosphoro-2AI)-rCrCrArCrCrGrCrArUrUrCrCrGrCrA
LS2-C	RNA	(5'-phosphoro-2AI)-rCrCrCrArCrCrGrCrArUrUrCrCrGrCrA
LS2-G	RNA	(5'-phosphoro-2AI)-rGrCrCrArCrCrGrCrArUrUrCrCrGrCrA
LS2-U	RNA	(5'-phosphoro-2AI)-rUrCrCrArCrCrGrCrArUrUrCrCrGrCrA
LT1	RNA	rGrCrGrGrUrGrGrUrCrCrUrUrArGrCrC
LT1+1	RNA	rUrGrCrGrGrUrGrGrUrCrCrUrUrArGrCrC
LT1+2	RNA	rArUrGrCrGrGrUrGrGrUrCrCrUrUrArGrCrC
LT1+3	RNA	rArArUrGrCrGrGrUrGrGrUrCrCrUrUrArGrCrC
LT1+4	RNA	rGrArArUrGrCrGrGrUrGrGrUrCrCrUrUrArGrCrC
LT1+5	RNA	rGrGrArArUrGrCrGrGrUrGrGrUrCrCrUrUrArGrCrC
LT1+6	RNA	rCrGrGrArArUrGrCrGrGrUrGrGrUrCrCrUrUrArGrCrC
LT1+7	RNA	rGrCrGrGrArArUrGrCrGrGrUrGrGrUrCrCrUrUrArGrCrC
LT1+8	RNA	rUrGrCrGrGrArArUrGrCrGrGrUrGrGrUrCrCrUrUrArGrCrC
LT2	DNA	GCGGUGGUCCUUAGCC
ME1	DNA	mCmCTTAGCCAAAAAAGGACAGCGAGCTC
ME2	DNA	mCmCTTAGCCAAAAAAGGACAGCGTGG
ME3	DNA	mCmC TTAGCCAAAAAAGGACAGCGGATTTATCC
ME4	DNA	mCmCTTAGCCAAAAAAGGACAGCGCG
ME5	DNA	same as ME4

ME6	DNA	mCmCTTAGCCAAAAAAGGACAGCGAGACC
ME7	DNA	mCmCTTAGCCAAAAAAGGACAGCGCC
ME8	DNA	mCmCTTAGCCAAAAAAGGACAGCGGG
ME9	DNA	mCmC TTAGCCAAAAAAGGACAGCGCC
ME10	DNA	mCmC TTAGCCAAAAAAGGACAGCGCAG
ME1-1	DNA	mCmUT AGC CAA AAA AGG ACA GCG AGC TCA GTC
ME1-2	DNA	mUmUA GCC AAA AAA GGA CAG CGA GCT CAG TCT G
ME1-A	DNA	mUmCTTAGCCAAAAAAGGACAGCGAGCTC
ME1-C	DNA	mGmCTTAGCCAAAAAAGGACAGCGAGCTC
ME1-U	DNA	mAmCTTAGCCAAAAAAGGACAGCGAGCTC
OH1	RNA	rArUrUrCrCrGrCrA
PCR1	DNA	CGGTAGGTCCCTTAGCCAAAAAAGG
PCR2	DNA	TAATACGACTCACTATAGACTCACTGACACAGATCCACTCAC
PS1	DNA	GTTCAGAGTTCTACAGTCCGACGATCCGGTAGGTCCCTTAGCCAAA AAAGG
PS2	DNA	AGACGTGTGCTCTTCCGATCTACTCACTGACACAGATCCACTCAC
PS3	DNA	GTTCAGAGTTCTACAGTCCGACGATC
PS4	DNA	AGACGTGTGCTCTTCCGATCT
RQ1	DNA	GTGCGGAATGCGGTGGT
RQ2	DNA	GCCTTAGCCAAAAAAGGACAGCG
RT1	DNA	CGGTAGGTCCCTTAGCCAAAAAAGGACAGCG
RT2	DNA	GTGCGGAATGCGGTGGTCCTT
SL1	DNA	CGGTAGGTCCCTTAGCCAAAAAAGGACAGCGNNNNNNN NNNNNNNNNNNNNNNNNNNNNNNNNNNNNNNNNNCGCT GTCCGTGAGTGGATCTGTGTCAGTGAGTCTATAGTGAGT CGTATTA
TT1	DNA	mGmGACAGCGAGCTCAGTCTGC
TT2	DNA	mGmGACAGCGTGGTTCCGC
TT3	DNA	mGmGACAGCGGATTTATCCCAATTCTCTG
TT4	DNA	mGmGACAGCGCGGCTTCC
TT5	DNA	mGmGACAGCGCGCTTAGGCTTAC
TT6	DNA	mGmGACAGCGAGACCGCC
TT7	DNA	mGmGACAGCGCCACTTCACG
TT8	DNA	mGmGACAGCGGGGTGAGCAATTAC
TT9	DNA	mGmGACAGCGCCGTTTCCTC
TT10	DNA	mGmGACAGCGCAGTTCTGTTCTGC

Table S3. Sequences used in this study.

SI REFERENCES

1. T. Walton, L. Pazienza, J. W. Szostak, Template-Directed Catalysis of a Multistep Reaction Pathway for Nonenzymatic RNA Primer Extension. *Biochemistry* **58**, 755–762 (2019).

2. I. A. Murray, S. K. Stickel, R. J. Roberts, Sequence-specific cleavage of RNA by Type II restriction enzymes. *Nucleic Acids Res*. **38**, 8257–8268 (2010).

3. C. Kao, M. Zheng, S. Rüdisser, A simple and efficient method to reduce nontemplated nucleotide addition at the 3′ terminus of RNAs transcribed by T7 RNA polymerase. *RNA* **5**, 1268–1272 (1999).

4. D. H. Matthews, *et al.*, Incorporating chemical modification constraints into a dynamic programming algorithm for prediction of RNA secondary structure. *Proc. Natl. Acad. Sci. U.S.A.* **101**, 7287–7292 (2004).

5. G. M. Rice, S. Busan, F. Karabiber, O. V. Favorov, K. M. Weeks, RNA SHAPE Analysis of Small RNAs and Riboswitches. *Methods Enzymol.* **549**, 165–187 (2014).

6. K. A. Wilkinson, E. J. Merino, K. M. Weeks, Selective 2'-hydroxyl acylation analyzed by primer extension (SHAPE): quantitative RNA structure analysis at single nucleotide resolution. *Nat. Protoc.* **1**, 1610–1616 (2006).

7. T. Walton, J. W. Szostak, A Kinetic Model of Nonenzymatic RNA Polymerization by Cytidine-5'-phosphoro-2-aminoimidazolide. *Biochemistry* **56**, 5739–5747 (2017).
